# Colorectal cancer screening preferences among physicians and individuals at average risk: A discrete choice experiment

**DOI:** 10.1002/cam4.4678

**Published:** 2022-03-21

**Authors:** Sebastian Heidenreich, Lila J. Finney Rutten, Lesley‐Ann Miller‐Wilson, Cecilia Jimenez‐Moreno, Gin Nie Chua, Deborah A. Fisher

**Affiliations:** ^1^ Evidera London UK; ^2^ Division of Epidemiology, Department of Quantitative Health Sciences Mayo Clinic Rochester Minnesota USA; ^3^ Exact Sciences Corporation Madison Wisconsin USA; ^4^ Duke Clinical Research Institute Durham North Carolina USA

**Keywords:** colonoscopy, colorectal cancer, colorectal neoplasms, early detection of cancer, patient preference, physicians, screening

## Abstract

**Background:**

Guidelines include several options for average‐risk colorectal cancer (CRC) screening that vary in aspects such as invasiveness, recommended frequency, and precision. Thus, patient and provider preferences can help identify an appropriate screening strategy. This study elicited CRC screening preferences of physicians and individuals at average risk for CRC (IAR).

**Methods:**

IAR aged 45–75 years and licensed physicians (primary care or gastroenterology) completed an online discrete choice experiment (DCE). Participants were recruited from representative access panels in the US. Within the DCE, participants traded off preferences between screening type, screening frequency, true‐positive, true‐negative, and adenoma true positive (physicians only). A mixed logit model was used to obtain predicted choice probabilities for colonoscopy, multi‐target stool DNA (mt‐sDNA), fecal immunochemical test (FIT), and methylated septin 9 (mSEPT9) blood test.

**Results:**

Preferences of IAR and physicians were affected by screening precision and screening type. IAR also valued more regular screening. Physicians preferred colonoscopy (96.8%) over mt‐sDNA (2.8%; *p* < 0.001), FIT (0.3%; *p* < 0.001) and mSEPT9 blood test (0.1%; *p* < 0.01). IAR preferred mt‐sDNA (38.8%) over colonoscopy (32.5%; *p* < 0.001), FIT (19.2%; *p* < 0.001), and mSEPT9 blood test (9.4%; *p* < 0.001). IAR naïve to screening preferred non‐invasive screening (*p* < 0.001), while the opposite was found for those who previously underwent colonoscopy or sigmoidoscopy.

**Conclusions:**

While physicians overwhelmingly preferred colonoscopy, preferences of IAR were heterogenous, with mt‐sDNA being most frequently preferred on average. Offering choices in addition to colonoscopy could improve CRC screening uptake among IAR.

This study used a discrete choice experiment in the US to elicit preferences of physicians and individuals at average risk for colorectal cancer screening modalities and their characteristics.

## Graphical abstract

Physicians preferred colonoscopy over multitarget stool DNA test (mt‐sDNA; *p* < 0.001), fecal immunochemical test (FIT;*p* < 0.001), and mSEPT9 blood test (*p* < 0.01). Individuals at average risk preferred mt‐sDNA over colonoscopy (*p* < 0.001), FIT (*p* < 0.001), and mSEPT9 blood test (*p* < 0.001). Individuals naïve to screening preferred (*p* < 0.001) non‐invasive screening, while the opposite was found for those who previously underwent colonoscopy or sigmoidoscopy.

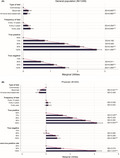

## 1 INTRODUCTION

Although colorectal cancer (CRC) is treatable if detected at an early stage, it remains the second leading cause of cancer death in the US.^1^, ^2^ The US Preventive Services Task Force (USPSTF) has traditionally recommended CRC screening for adults aged 50–75 years who have an average risk for CRC.^3^, ^4^ However, the USPSTF now also recommends screening from 45 to 49 years of age,^3^ in line with recommendations of the American Cancer Society (45 years and older)^5^ since there is an expected benefit from screening this younger age group. Despite guidance and recommendations, overall screening rates remain below the 80% target rate endorsed by The National Colorectal Cancer Round Table.^6^ Roughly one‐third of eligible adults in the US are not up‐to‐date with CRC screening or have not been screened for CRC, and adherence to screening recommendations varies between sub‐groups in the population.^7^, ^8^, ^9^, ^10^

Several CRC screening options are available, including the fecal immunochemical test (FIT), the multitarget stool DNA (mt‐sDNA) test, sigmoidoscopy, colonoscopy, the fecal occult blood test (FOBT), and a blood test that detects methylated septin 9 (mSEPT9).^11^ These screening options differ in precision (i.e., sensitivity and specificity), burden to patients, risks of complications, and cost. Colonoscopy is the CRC screening method for preventing CRC recommended by the American College of Gastroenterology and the American Gastroenterology Association.^12^, ^13^ Alternatives (e.g., stool‐based tests, flexible sigmoidoscopy, CT colonography) carry the limitation that a positive test result requires a follow‐up colonoscopy.^12^

Research suggests that patients are more likely to undergo CRC screening if it is recommended by their primary care provider.^14^, ^15^ However, patients may prefer a different screening modality than the one recommended,^16^, ^17^, ^18^ and their adherence may be influenced by perceptions and preferences.^16^, ^18^, ^19^, ^20^ For example, although colonoscopy is the most frequently recommended option, recommending colonoscopy alone may reduce adherence to CRC screening, especially among racial and ethnic minorities. Accordingly, it has been advocated that among available options, the best screening method is the one that a patient is willing to undertake.^3^ Thus, healthcare providers are encouraged to engage patients in shared decision‐making that integrates clinical and scientific evidence of CRC screening approaches with patients' preferences, needs, and values.^21^, ^22^, ^23^ Research about the specific screening test attributes that influence individuals' and physicians' preferences may help guide information exchange and collaborative decision‐making and thereby improve CRC screening uptake.

This study elicited preferences of individuals at average risk (IAR) and physicians for colonoscopy, FIT, mt‐sDNA test, and mSEPT9 blood test. It also quantified the relative importance these groups place on different attributes of CRC screening methods to provide a foundation for discussions about CRC screening options and increase uptake in the population.

## 2 METHODS

### 2.1 Study overview

A cross‐sectional online survey was conducted in the US between November 18, 2020, and February 8, 2021, in IAR for CRC and two groups of physicians (i.e., primary care physicians [PCPs] and gastroenterologists). The survey included a discrete choice experiment (DCE) to elicit preferences of IAR and physicians regarding CRC screening. A graphical overview of the DCE development and study conduct is given in Figure S1. DCEs are well established within medical and health policy research and elicit preferences by asking participants to repeatedly choose between hypothetical healthcare alternatives that are described by a common set of attributes.^24^, ^25^, ^26^ The hypothetical nature of DCEs allows the levels that the attributes take to be varied, such that respondents are faced with trade‐offs. This experimental setup avoids the confounding of attributes that naturally occurs with real‐world data.

Before starting the DCE, participants were provided an overview of the purpose and structure of the online survey; an introduction to currently available CRC screening options; and were introduced to the DCE attributes, attribute levels, and the overall choice context. This introduction included denominator training for true‐positive and true‐negative rates and quizzes with feedback to improve participants' understanding. After completing the DCE, IAR were asked to answer health literacy questions and complete an attitudinal questionnaire in which they denoted their level of agreement with statements about CRC and screening. Physicians were asked about their perceived need for improvement in aspects related to CRC screening strategies, such as burden for patients, patient willingness to be screened, patient awareness of CRC screening, and the current standard age to start recommending screening. All participants were also required to answer sociodemographic questions. The IAR and physician surveys are provided in File S1.

The study was reviewed and approved by the ethics and institutional review board (E&I study # 20116 ‐ 01C) and conducted in line with relevant guidelines of the International Society for Pharmacoeconomics and Outcomes Research (ISPOR).^27^, ^28^, ^29^ All participants provided online consent before participating in the survey.

### 2.2 Participants

All participants were recruited by non‐probabilistic sampling from commercially managed online access panels (i.e., a large database of individuals with profiles that pre‐consented to being contacted for research). The panel composition from which participants were recruited was representative of the US general population. Potentially eligible participants were invited to participate via e‐mails using standardized recruitment templates, which contained a web link to the survey.

To participate in the study, IAR had to self‐report that they were 45–75 years of age; resided in the US; and could read, speak, and understand English. Respondents were excluded if they self‐reported personal or family history of CRC or personal history of inflammatory bowel disease or other gastrointestinal disorders. To ensure a representative sample, recruitment quotas were used based on the gender, race, and ethnicity composition of the 2019 US Census estimates^30^ (see Table S1). However, individuals aged 45–49 years were strategically oversampled (quota, 30%–40%) to facilitate subgroup analysis for this age group.

Physicians (recruited from a separate access panel) could only participate in the survey if they were an English‐speaking registered PCP or gastroenterologist with a current US practice and had recommended or performed ≥1 CRC screening in the month prior to the study. The quota for physician representation was 50% PCPs and 50% gastroenterologists.

### 2.3 DCE design

**FIGURE 1:**
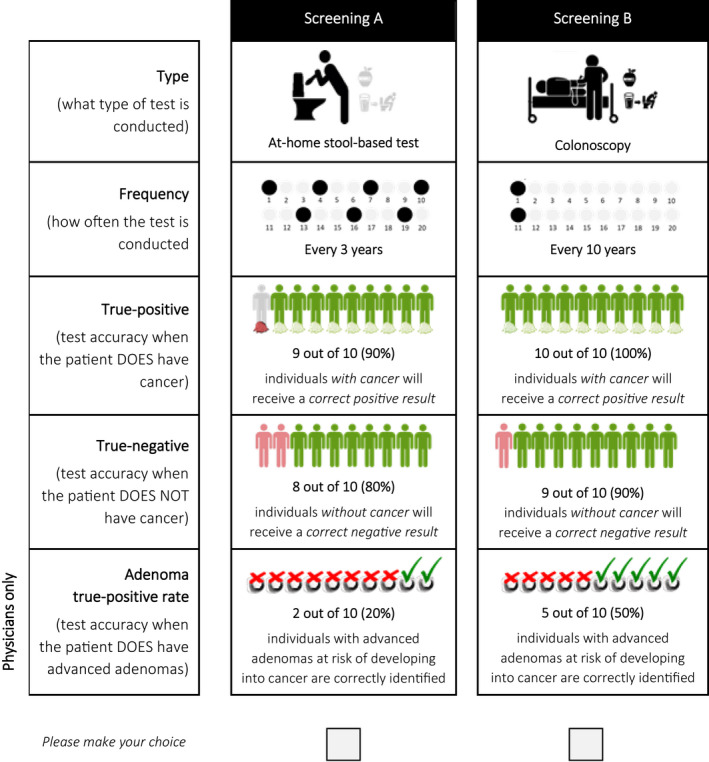
Example discrete choice experiment (DCE) task between two colorectal cancer screening options described by five screening test attributes. Example is from the DCE for physicians

The CRC screening attributes and levels used in the DCE were informed by a targeted literature review, clinical trial outcomes, and a workshop with clinical experts. Five key attributes were selected: (1) screening type, (2) screening frequency, (3) true‐positive rate, (4) true‐negative rate, and (5) for physicians the advanced adenoma true‐positive rate (Table 1). Within the DCE, all participants were asked to repeatedly choose between two screening scenarios labeled A and B that were described by the included attributes. An example choice task is presented in Figure 1. Separate D‐efficient experimental DCE designs that varied the levels of the CRC screening attributes between choice tasks were created for IAR and physicians.^31^ The design for IAR had 40 choice tasks that were split into four blocks of 10 experimental tasks. In contrast, the design for physicians had 36 choice tasks that were split into three blocks of 12 experimental tasks. All participants were randomly assigned to a block, and choice tasks were randomized to avoid ordering effects.

**TABLE 1:** DCE attributes and levels

Attribute	Description	Levels
Type	*Colonoscopy:* This test is conducted in a clinic or hospital. A trained physician will insert a thin, flexible tube with a camera into the rectum and inspect the colon. Sedation is required. You will be required to limit your food intake to specific foods (low‐fiber) a few days before and to fluids only the day before. Preparation also includes drinking a laxative (about half a gallon) the evening before the test, and the morning of the test. The laxative will cause diarrhea to clear the bowels and can in some cases cause dizziness, nausea, or vomiting.	(1) Colonoscopy (2) At‐home stool‐based test (3) Blood test
	*At‐home stool‐based test:* A test kit will be given to the patients. The kit includes all materials required for taking a stool sample and posting it to a dedicated laboratory. The stool will be collected in a sample container and sealed in a bag, before returning it for testing. Some tests may require adding provided chemicals to the stool sample in the sample container. In the case of a positive finding, meaning potential cancer was identified, a follow‐up colonoscopy should be undertaken to confirm the finding.	
	*Blood test:* A blood sample is taken by a healthcare professional in a local clinic or hospital. The healthcare professional collects a routine sample of blood by inserting a needle into a vein in the arm. The blood will then be analyzed in a laboratory. In the case of a positive finding, meaning a potential cancer was identified, a follow‐up colonoscopy should be undertaken to confirm the finding.	
Frequency^a^	This is how often (in years) the screening test is supposed to be conducted according to medical guidelines. Screening more often than what is recommended by the guidelines may not necessarily increase the chance of finding a potential cancer.	(1) Every year (2) Every 3 years (3) Every 10 years
True‐positive^b^	The true‐positive rate is the proportion of tested individuals with cancer, who are correctly identified by the test as having cancer. Thus, the higher the true‐positive rate, the higher is the chance of finding cancer, if it exists. For example, a true‐positive rate of 9 out of 10 (90%) means that out of 10 tested individuals with cancer, 9 (90%) are correctly identified as having cancer. The remaining individual (10%) with cancer is incorrectly identified as not having cancer, despite actually having cancer (false negative).	(1) 6 out of 10 (60%) (2) 7 out of 10 (70%) (3) 8 out of 10 (80%) (4) 9 out of 10 (90%) (5) 10 out of 10 (100%)
True‐negative	The true‐negative rate is the proportion of tested individuals with no cancer, who are correctly identified by the test as not having cancer. A high true‐negative rate increases the risk of unnecessary procedures (e.g., additional follow‐up colonoscopies). For example, a true‐negative rate of 9 out of 10 (90%) means that out of 10 tested individuals with no cancer, 9 (90%) are correctly told they do not have cancer. The remaining individual (10%) without cancer is incorrectly identified as having cancer despite being cancer‐free (false positive).	(1) 7 out of 10 (70%) (2) 8 out of 10 (80%) (3) 9 out of 10 (90%) (4) 10 out of 10 (100%)
Adenoma true‐positive rate^c^ (physicians only)	Adenoma true‐positive rate refers to correctly identifying those with polyps that are at risk of developing cancer in the future. For example, an adenoma true‐positive rate of 2 out of 10 (20%) means that out of 10 polyps that are at risk of developing into cancer in the future, 2 (20%) were correctly identified by the test (true positives), and 8 (80%) were incorrectly identified (false negatives).	(1) 2 out of 10 (20%) (2) 5 out of 10 (50%) (3) 10 out of 10 (100%)

^a^ ‘Every year’ not available for colonoscopy choice option.

^b^ ‘70%’ not available for colonoscopy choice option.

^c^ ‘20%’ not available for colonoscopy choice option.

Design constraints were imposed to mitigate the risk of implausible choice tasks. Specifically, colonoscopy was never paired with an annual frequency or a true‐positive rate <80%, and the true‐positive and true‐negative rates were never 100% at the same time.

In addition, each participant completed two non‐experimental choice tasks to assess internal validity: choice task #5 (as seen by the participants) was repeated as the second‐to‐last choice task to test the stability of participants' preferences, and a dominance test featuring a clearly superior choice was included as the final task to test participants' level of engagement with the survey.

### 2.4 DCE pilot testing

To test the robustness of the developed DCE, quantitative and qualitative pilot tests were conducted. The quantitative pilot (File S1) assessed the expected data quality and preference‐relevance of included attribute levels. A total of 202 members of the US population at average risk for CRC were recruited, in addition to 50 PCPs and 50 gastroenterologists.

The qualitative pilot (File S3) determined if the chosen attributes, levels, and the format of presentation were understood by participants, and if screening test attributes were traded across alternatives. All pilot participants were asked to explain key concepts in their own words to inform iterative updates to the survey. For instance, misunderstandings about true‐positive and true‐negative rates informed the development of training on denominators, with successful knowledge transfer assessed using a quiz.

To improve the precision of the data analysis, preference information obtained during the quantitative pilot was used to update the experimental designs using Bayesian priors.^31^

### 2.5 Statistical analysis

Sample characteristics were summarized using descriptive statistics. DCE preference data were analyzed following random utility theory by representing participants' preferences by a rank‐order preserving continuous utility function with an extreme‐value distributed error.^32^ Effects of changes in each attribute (i.e., marginal utilities) on screening utility were estimated using a mixed logit (MXL) model that accounted for heterogeneity in participants' preferences (File S4). The estimated utility function was dummy coded, such that marginal utilities were estimated relative to a reference level. An alternative‐specific constant was added to account for left–right bias. All marginal utilities were assumed to follow a normal distribution; thus, population mean marginal utilities and corresponding standard deviations (SDs) were estimated. A significant SD denoted the presence of preference heterogeneity.

Three behavioral outputs were obtained from the model estimates. First, relative attribute importance (RAI) scores were calculated as the normalized effect on utility of moving from the least‐preferred to the most‐preferred level within each attribute. Hence, RAI scores measured the maximum percentage change in utility caused by changes in each screening test attribute. Standard errors were obtained using the delta method.^33^ Second, average partial effects (APEs) were estimated as the average effect of changes in each attribute on the likelihood of a screening test being preferred over an alternative. APEs were obtained over the full factorial combination of attribute levels across two alternatives and calculated using the estimated mean marginal utilities. Third, predicted choice probabilities (PrCPs) were calculated to compare the desirability of profiles representing colonoscopy, mt‐sDNA test, FIT, and mSEPT9 blood test. PrCPs denoted the likelihood of a screening option being preferred over all alternatives. Profiles of screening options were informed by the USPSTF and pivotal clinical trials (Table 2).^34^

**TABLE 2:** Preference probabilities

Attribute	FIT^a^	mt‐sDNA test^a^	Blood test^b^	Colonoscopy^c^
Frequency	Every 1 year	Every 3 years	Every 1 year	Every 10 years
True‐positive	73.8%	92.3%	72%	95%
True‐negative	94.9%	86.6%	81%	86%
Adenoma true‐positive rate	23.8%	42.4%	11%	95%

Abbreviations: FIT, fecal immunochemical test; mt‐sDNA, multitarget stool DNA.

^a^ Reference values: Imperiale et al. 2014.^39^

^b^ Reference values: Johnson et al. 2014.^41^, ^42^

^c^ Reference values: Knudsen et al. 2016.^34^

Subgroup analyses were conducted in the IAR cohort to assess differences in screening experience, age, and race, and in the physician cohort to assess differences between gastroenterologists and PCPs. Interaction effects with *p*‐values <0.1 were included in the final models.

The generalizability of the preference estimates to the wider US population of IAR was assessed by re‐weighting the sample to match the composition of the 2019 US Census estimates^30^ and comparing estimates to the unweighted MXL model. Model estimates were compared using a *z*‐test.

Quantitative analysis was conducted using Stata 15.1 (StataCorp LLC).

## 3 RESULTS

### 3.1 Disposition and sample characteristics

#### 3.1.1 Characteristics of IAR cohort

Out of the 6882 IAR who clicked on the survey link, 2214 (32.2%) were screened into the study, and of these 1585 (71.6%) provided informed consent and started the survey (Figure S2). In total, 1249 (56.4%) of screened IAR completed the survey (including the quantitative pilot sample). The mean age of the IAR cohort was 58.9 ± 9.1 years, 46% were male, and 82% were Caucasian (Table 3). Most IAR had adequate health literacy (*n* = 1223, 97.9%) and adequate health numeracy (*n* = 1104, 88.4%). One‐third of IAR were retired (*n* = 412; 33.0%), with most of the remainder employed full‐time (*n* = 491; 39.3%) or part‐time (*n* = 132; 10.6%). Most IAR reported their health to be good (*n* = 661; 53%) or very good (*n* = 279; 22%). Overall, the sample composition did not significantly differ from the 2019 US Census estimates in terms of gender (*p* > 0.05) or race (*p* > 0.05). General attitudes and concepts of IAR toward CRC and screening are described in File S5 and Figure S3.

**TABLE 3:** Characteristics of individuals at average risk (IAR; *N* = 1249) and physician cohorts (*N* = 400)

Characteristics	Value
*IAR*	
Age, mean ± SD, years	58.9 ± 9.1
Gender	
Female	670 (53.6)
Male	578 (46.3)
Other	1 (0.1)
Race, *n* (%)	
Caucasian	1021 (81.7)
African American	105 (8.4)
Asian American	45 (3.6)
Other	78 (6.2)
Employment status, *n* (%)	
Full‐time	491 (39.3)
Part‐time	132 (10.6)
Retired	412 (33.0)
Unemployed	88 (7.0)
Disability	70 (5.6)
*Physicians*	
Age, mean ± SD, years	53.4 ± 10.5
Gender	
Female	82 (20.5)
Male	316 (79.0)
Other	2 (0.4)
Medical specialty, *n* (%)	
Primary care physician	200 (50.0)
Gastroenterologist	200 (50.0)
Duration practicing medicine, mean ± SD, years	21.7 ± 9.3
Duration recommending CRC screening tests, mean ± SD, years	21.5 (9.4)
Type of clinical practice, %	
Single‐specialty group	43.8
Multi‐specialty group	26.8
Solo practice	17.0
Academic system or hospital	12.0
Other	0.5

Abbreviations: CRC, colorectal cancer; SD, standard deviation.

#### 3.1.2 Characteristics of physician cohort

Of the physicians who clicked on the survey link (*n* = 567), 441 (77.8%) were screened into the study and 420 (74.1%) provided informed consent and started the survey (Figure S4). A total of 400 physicians completed the survey (*n* = 200 gastroenterologists and *n* = 200 PCPs). The mean age of the physician cohort was 53.4 ± 10.5 years and 79.0% (*n* = 316) were male (Table 3). Characteristics of physician practice settings and physician experience as well as physicians' perceived needs for improving specific aspects of CRC screening are described in File S6 and Figure S5.

### 3.2 Drivers of screening preferences

#### 3.2.1 Internal validity of the preference data

The internal validity assessments suggested that participants were trading off between attributes when choosing their preferred screening alternative in the DCE. Specifically, 93.7% (*n* = 1170) of the IAR cohort and 97.8% (*n* = 391) of the physician cohort passed the choice dominance test, and 82.1% (*n* = 1025) of the IAR cohort and 90.0% (*n* = 360) of the physician cohort passed the stability test (Table S2). Furthermore, 99.1% (*n* = 1238) of IAR and 100.0% (*n* = 400) of physicians varied their choices among the screening options in the DCE. Overall, the internal validity was comparable or higher than in other health‐related DCE studies in the literature.^29^

#### 3.2.2 Model Performance

**FIGURE 2:**
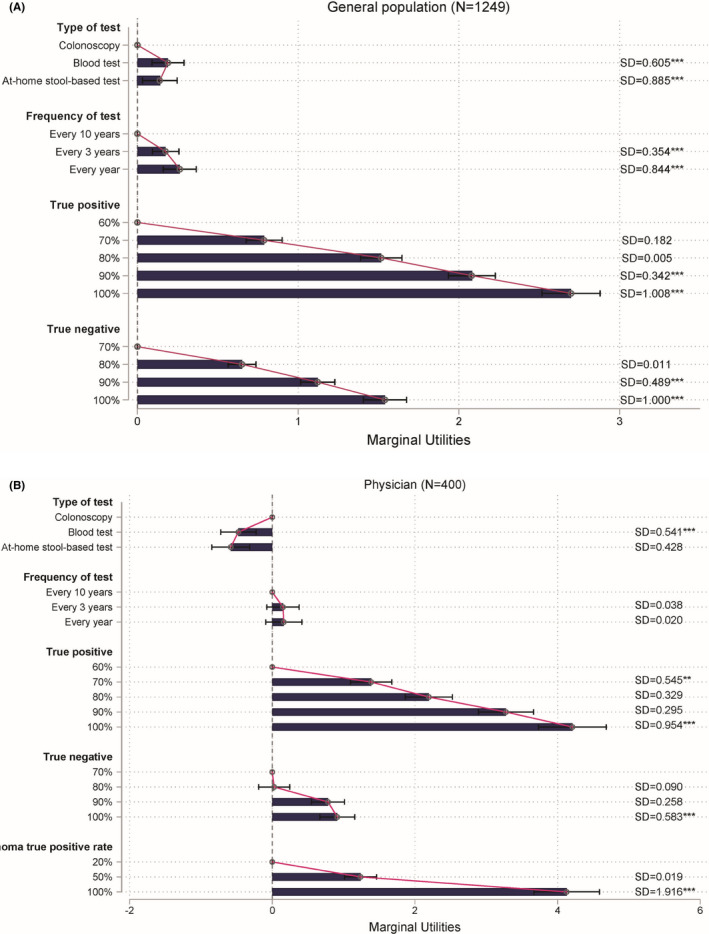
MXL model estimates among (A) individuals at average risk (*N* = 1249) and (B) physicians (*N* = 400) from the discrete choice experiment. Estimates denote how preferences are affected by deviating from the reference level (first level) in each attribute. Bars with a CI that does not cross zero capture a significant effect on preferences. The longer the bar, the larger is the impact on preferences. However, the relative magnitude of the difference between bars should not be interpreted due to the ordinal nature of underlying preferences and an arbitrary scale. For individuals at average risk (A), constant of left alternative was −0.094 (SE 0.026); final log‐likelihood at convergences: −6984; McFadden‐adjusted R^2^: 0.196; Bayesian information criterion: 14184. For physicians (B), constant of left alternative was −0.010 (SE 0.063); final log‐likelihood at convergences: −1456; McFadden‐adjusted *R* ^2^: 0.571; Bayesian information criterion: 3141. Estimation via maximum likelihood method: *p < 0.05; **p < 0.01; ***p < 0.001. Whiskers denote a 95% confidence interval

Estimates from the MXL model for the IAR sample are provided in Figure 2A and Tables S3 and S4, and for the physician sample in Figure 2B and Table S5. The presented specifications were preferred by a likelihood ratio test over a model without screening test attributes (*p* < 0.001), suggesting that all screening test attributes jointly explain choices made in the DCE. Based on Bayesian Information Criteria (IAR: 14,184 vs. 14,406; physicians: 3141 vs 3342), the estimated MXL model was preferred over a multinomial logit model that did not account for preference heterogeneity, suggesting that preferences varied within each cohort.

#### 3.2.3 Utilities, relative attribute importance, and trade‐offs

In the IAR cohort, blood tests (*p* < 0.001) and at‐home stool‐based tests (*p* = 0.012) were significantly preferred over colonoscopy (Figure 2A). In contrast, physicians preferred colonoscopy significantly over blood tests (*p* < 0.001) and at‐home stool‐based tests (*p* < 0.001; Figure 2B). While IAR significantly valued more regular screening (i.e., every 3 years or every year, for blood test and at‐home stool‐based test only; *p* < 0.001), physicians' preferences were not significantly affected by the screening frequency. Both IAR and physicians preferred higher true‐positive and true‐negative rates (*p* < 0.001). In addition, physicians valued higher true‐positive rates for detecting advanced adenoma (*p* < 0.001).

Although most included attributes were valued by both IAR and physicians, their relative importance differed between populations (Figure S6). The true‐positive rate was the largest driver of preferences for both IAR (RAI score = 57.5%) and physicians (RAI score = 42.1%). Physicians also considered the true‐positive rate for advanced adenoma as being of comparable importance (RAI score = 41.3%) to the true‐positive rate for detecting CRC. True‐negative rates were less important than true‐positive rates to both IAR (RAI score = 32.8%) and physicians (RAI score = 9.1%). Despite being significant drivers of preferences, IAR placed less importance on the frequency of CRC screening (RAI score = 5.6%) and test type (RAI score = 4.1%) than screening precision. Similarly, physicians placed less importance on the test type (RAI = 5.8%) and frequency (RAI = 1.6%).

APE estimates suggest that physicians and IAR were willing to make trade‐offs between included attributes (Table S6). For example, IAR were willing to accept a reduction of the true‐positive rate from 100% to 90% (−10.6%) if that was compensated by an increase in the true‐negative rate from 80% to 100% (+16.0%). Similarly, IAR were willing to accept a reduction in the true‐negative rate from 90% to 80% (−8.6%) and screening frequency from every year to every 3 years (−1.6%), if the true‐positive rate was increased from 80% to 90% (−10.8%). APEs for physicians suggest that they were willing to accept a reduction in the adenoma true‐positive rate from 70% to 80% (−6.4%) in exchange for an increase in the cancer true‐positive rate from 80% to 90% (+12.2%).

Subgroup analyses revealed differences in screening test preferences by age group and race in the IAR cohort (Figures S7–S9; Table S7). IAR aged 65–75 years placed significantly more importance on true‐positive (*p* < 0.05) and true‐negative (*p* < 0.05) rates than did IAR aged 45–49 years (Table 4). Preferences of non‐White IAR were significantly (*p* < 0.001) less affected by true‐positive rate than preferences of White respondents.

**TABLE 4:** Differences between subgroups in individuals at average risk (*N* = 1249)

	Age			Race		Screening Experience^c^		
	45–49 years	50–64 years	65–75 years	White	Non‐White	None	Non‐invasive	Invasive
Type (versus colonoscopy)^a^	Reference			Reference		Reference		
Blood test		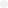	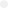		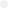		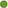	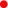
At‐home stool‐based test		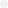	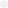		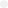		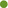	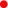
Frequency^b^		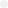	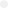		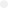		‐	‐
True positive^b^		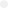	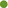		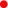		‐	‐
True negative^b^		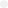	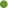		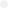		‐	‐

*Note*: Green circles represent results > than the reference; red circles represent results < than the reference.

^a^ Based on the coefficient estimate.

^b^ Based on relative importance scores.

^c^ Only colonoscopy and flexible sigmoidoscopy were considered as invasive.

### 3.3 Comparison of screening methods

**FIGURE 3:**
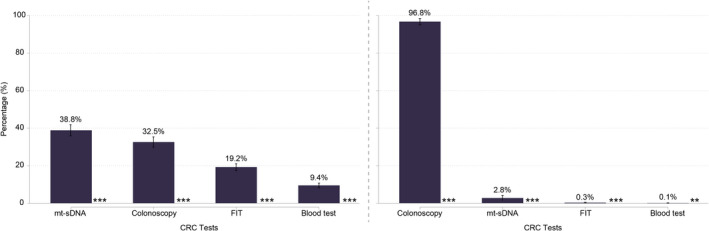
Screening preferences in IAR and physician cohorts based on the discrete choice experiment. This figure shows predicted choice probabilities (PrCPs) for different screening profiles. PrCPs quantify the likelihood of each profile being preferred over all alternatives. Profiles are sorted by preferential order and stars indicate if one profile is preferred over the next ranked profile. Whiskers denote corresponding 95% confidence interval. FIT, fecal immunochemical test; mt‐sDNA, multitarget stool DNA. *p* < 0.05; **p < 0.01; ***p < 0.001

Although both IAR and physicians placed most importance on the precision of CRC screening, preferences differed for the overall profiles of the alternative screening tests, i.e., when accounting for the combination of multiple attributes (Figure 3). IAR preferred mt‐sDNA over colonoscopy (PrCP mt‐sDNA = 38.8%, PrCP colonoscopy = 32.5%; *p* < 0.001), whereas physicians preferred colonoscopy (PrCP colonoscopy = 96.8%, PrCP mt‐sDNA 2.8%; *p* < 0.001). Despite this disagreement about the preferred screening method, mt‐sDNA and colonoscopy were both significantly (*p* < 0.001) preferred over FIT by IAR (PrCP FIT = 19.2%) and physicians (PrCP FIT = 0.3%). Finally, FIT was significantly (*p* < 0.001) preferred over mSEPT9 blood test, by IAR (PrCP mSEPT9 blood test = 9.4%) and physicians (PrCP mSEPT9 blood test = 0.1%).

**FIGURE 4:**
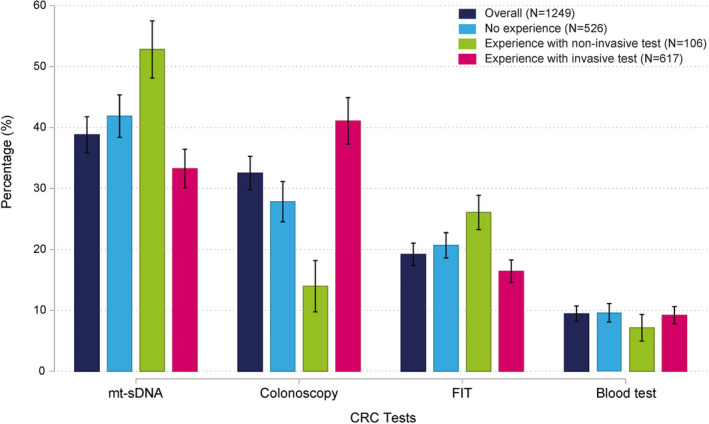
Test preferences of the IAR cohort based on previous screening experience. This figure shows predicted choice probabilities (PrCPs) for different screening profiles. PrCPs quantify the likelihood of each profile being preferred over all alternatives. Blood and stool tests were considered non‐invasive; colonoscopy and flexible sigmoidoscopy were considered as invasive. Whiskers denote corresponding 95% confidence interval. FIT, fecal immunochemical test; mt‐sDNA, multitarget stool DNA

Screening test preferences of IAR differed based on their prior screening experience **(**Figure 4). Most notably, IAR who previously underwent invasive screening (i.e., colonoscopy or sigmoidoscopy) preferred colonoscopy over non‐invasive treatments (*p* < 0.001), whereas unscreened IAR and those who previously only used non‐invasive screening preferred to avoid colonoscopy (*p* < 0.001). In addition, previously screened IAR who were naïve to invasive methods formed the only subgroup who also significantly (*p* < 0.001) preferred FIT over colonoscopy. Screening preferences for other subgroups were consistent with the population averages.

### 3.4 Generalizability of findings

To explore the generalizability of the findings, the IAR sample was re‐weighted to match the composition of the 2019 US Census estimates^30^ in terms of gender, age, race, and ethnicity (Figure S10). The average difference between the estimated marginal utilities was less than 0.01%, and *z*‐tests indicated no significant differences between any estimate.

## 4 DISCUSSION

This study showed that CRC screening preferences of IAR and physicians were primarily driven by precision, as captured by the true‐positive rate, adenoma true‐positive rate (physicians only), and true‐negative rate. IAR also preferred screening intervals of less than every 10 years (for blood test and at‐home stool‐based test only), whereas physicians' choices were not significantly affected by screening frequency. Elicited preferences suggested that both physicians and IAR were willing to make trade‐offs between different CRC screening attributes.

Comparing profiles of different screening methods based on estimated preferences and clinical performance data revealed noticeable differences between preferences of physicians and IAR. Although IAR preferred mt‐sDNA over colonoscopy, physicians preferred colonoscopy. These findings are consistent with previously reported clinician preference for CRC screening by colonoscopy.^20^, ^35^ Additionally, these findings contribute to the evidence base suggesting that the common practice of recommending colonoscopy alone may result in suboptimal CRC screening uptake.^19^, ^20^, ^36^ Instead, offering a choice of CRC screening tests and recommending the use of tests that align with an individual's preferences may improve screening adherence. Providing IAR with clear information about the attributes of different screening options may support informed decision making.^37^

Preferences varied between individuals and subgroups. Notably, IAR without prior screening experience significantly preferred mt‐sDNA over colonoscopy. These findings suggest that non‐invasive screening could offer the first‐line path to screening for hesitant individuals who are screening naïve. Preference estimates also suggest that IAR would value a blood test alongside other available options, but only if future tests have higher true‐positive and true‐negative rates than existing FDA‐approved options.

To our knowledge, this was the first study that elicited CRC screening preferences among IAR and physicians for colonoscopy, FIT, mt‐sDNA, and mSEPT9 blood test. The strengths of the study included a rigorous instrument pre‐testing that focused on the clarity and understanding of relevant screening attributes to all participants. Furthermore, recruitment was based on quotas informed by the 2019 US Census to allow for testing of the generalizability of the findings. Some limitations should be acknowledged. The sample included fewer IAR who identified themselves as Hispanics or Latinos than the 2019 US Census estimates,^30^ and it is unknown if individuals who did not participate in the study would have had different preferences from those who participated. However, re‐weighting responses to ensure the sample composition matched the 2019 US Census estimates in terms of gender, age, race, and ethnicity suggested that estimates were generalizable to the wider population. Finally, the true‐positive rate of advanced adenomas was not included in the IAR population to avoid overburdening participants with more challenging concepts, which could have affected RAI estimates. However, a previous study found that few IAR placed high relative importance on adenomas.^38^

Previous studies have elicited preferences for CRC screening,^39^, ^40^ but they are limited in that they did not capture the most widely used screening tests or because they included attributes that are difficult to link to clinical data. Further research is needed to quantify the trade‐offs that physicians and patients are willing to make in choosing between screening test attributes.

## 5 CONCLUSIONS

This study demonstrated that CRC screening preferences differ between IAR and physicians. To improve CRC screening rates, IAR may need more information about the attributes of the various CRC screening options. Information exchange about the available screening options between IAR and clinicians may improve the alignment of clinician recommendations with individuals' preferences, thereby improving CRC screening adherence.

## CONFLICTS OF INTERESTS

SH, CJM, and GNC are employees of Evidera. Evidera received funding from Exact Sciences for conducting the work outlined in this manuscript. LAMW is an employee of Exact Sciences. DAF is a consultant for Exact Sciences. LFR is an employee of Mayo Clinic. Mayo Clinic has a consultancy contract with Exact Sciences.

## AUTHOR CONTRIBUTIONS

*Conceptualization*: SH, LAMW; *Methodology*: SH, CJM, LAMW, LFR, CJM, GNC, DAF; *Formal analysis*: GNC, SH; *Investigation*: SH, CJM, GNC; *Writing—Original Draft*: SH; *Writing—Review & Editing*: CJM, LAMW, LFR, CJM, GNC, DAF; *Project administration*: CJM; *Visualization*: GNC.

## IRB APPROVAL

The study protocol, consent forms, and all patient‐facing materials were reviewed and approved by an independent institutional review board (E&I study # 20116 ‐ 01C).

## Supporting information


Supplemental file 1

## Data Availability

The main survey data is available from the corresponding author on request.
