# Supplementary material for: Colorectal cancer screening preferences among physicians and individuals at average risk: A discrete choice experiment
Source: Cancer Med. 2022 Mar 21;11(16):3156–67. doi: 10.1002/cam4.4678 (PMC9385595; doi:10.1002/cam4.4678)
Supplement: Supplementary file 1 — Supplemental file 1 [file CAM4-11-3156-s001.docx]

# SUPPLEMENTAL MATERIALS

## Supplemental file 1: IAR and physician surveys

**Survey: Individuals at average risk**

| **Introduction** |
| --- |

Thank you for agreeing to participating in this survey. This study is conducted by the health research organization Evidera, on behalf of a sponsor to understand people’s perceptions of and preferences for colorectal cancer screening.

There are four sections in this survey, which will take approximately 30 minutes to complete:

- **Section 1** will introduce you to colorectal cancer and common colorectal cancer screening options that are currently available.
- **Section 2** will ask you to make several choices between alternative screening options. This is an established method that helps researchers understand what aspects of colorectal cancer screening matter to you.
- **Section 3** will ask you some general questions about colorectal cancer screening (e.g. your experience and opinion).
- **Section 4** will ask you about some sociodemographic information about yourself, including education level, employment status, marital status, lifestyle, and living situation.

Your responses will be *anonymous*, and your name will *never* be identified with any of your answers. All data will only be used for *medical research* purposes.

We are interested in understanding **your** perspective on colorectal cancer screening.

| **Section 1: About Colorectal Cancer and Screening** |
| --- |

**About Colorectal Cancer**

- Colorectal cancer is cancer that occurs in the colon (also known as the large bowel or large intestine) or rectum. Sometimes it is also called colon cancer or rectal cancer. The rectum is the passage that connects the colon to the anus. Colorectal cancer is the fourth most common cancer in the U.S. and both men and women have almost equal chances of getting it.

**Illustration of colon and rectum**


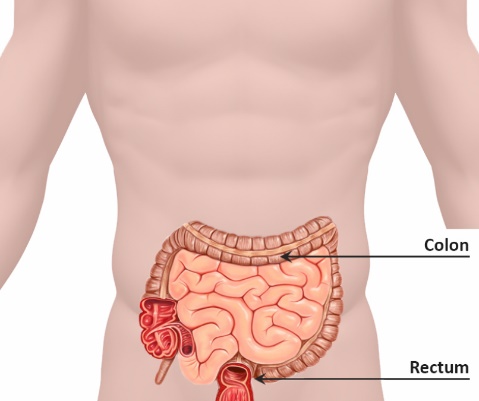


- Colorectal cancer typically starts as polyps (or adenomas) that develop into cancer. Polyps are lumps grown on the inner lining of the colon or rectum. Only some polyps will develop into cancer over time. While it is unclear if a polyp will become cancer, polyps larger than 1cm, or collections of polyps have an increased risk of becoming cancer. Once a polyp becomes cancer, it grows along and within the walls of the colon or rectum, and eventually spreads through the blood.
- There are several factors that make colorectal cancer more likely to develop. These include a family history of such cancer, being older than 50 years, smoking, being overweight, being physically inactive, regularly consuming moderate to large amounts of alcohol or certain foods (e.g. red or processed meat), smoking, consuming a low fiber diet (e.g. lack of fruits and vegetables), and having certain types of diseases (e.g. inflammatory bowel diseases).

**About Colorectal Cancer Screening**

- Several screening tests are available that can detect signs of actual or potential cancer in people, even if they do not experience any symptoms. Medical guidelines suggest that colorectal cancer screening should be conducted from an age of 50 years. Some evidence suggests that screening even younger people can help to prevent cancer early on.
- Three types of potential screening tests are colonoscopy, at-home stool-based test and blood test:
-
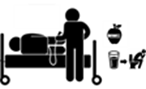
**Colonoscopy:** This test is conducted in a clinic or hospital. A trained physician will insert a thin, flexible tube with a camera into your rectum to inspect the colon. Individuals are usually sedated for this procedure. You will be required to limit your food intake to specific foods (low-fiber) a few days before, and to liquids only the day before the procedure. Preparation also includes drinking a laxative (about half a gallon) the evening before the test, and the morning of the test. The laxative will cause diarrhea to clear the bowels and can in some cases cause dizziness, nausea, or vomiting.
  - **At-home stool-based test**
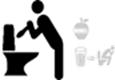
**:** You will be given a test-kit that includes all materials and equipment required for taking a stool sample. You will collect the stool in a sample container which is then sealed in a bag, before returning for processing. Test-kits may require adding provided chemicals to the stool sample in the sample container. In some cases, test-kits are delivered and picked-up from your home. In other cases, test-kits need to be picked up from and returned to a local clinic or hospital. In the case of a positive finding, a follow-up colonoscopy should be undertaken to confirm the finding - i.e. whether colorectal polyp(s) or cancer is present.
  - **
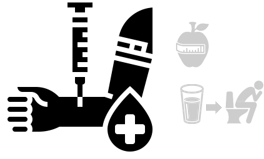
Blood test:** A blood sample is taken by a health care professional in a local clinic or hospital. The health care professional collects a routine sample of your blood by inserting a needle into a vein in your arm. The blood will then be analysed in a laboratory. In the case of a positive finding, a follow-up colonoscopy should be undertaken to confirm the finding - i.e. whether colorectal polyps(s) or cancer is present.
- Examples of other screening approaches include stool tests collected in a clinic and computed tomography (CT) which involves inserting a tube into the rectum that inflates the rectum and colon with air.

**About the Accuracy of Colorectal Cancer Screening**

Colorectal cancer screening tests differ in type (e.g. colonoscopy vs at-home stool-based test), but also in **accuracy**.

Please imagine that 1,000 people are screened for colorectal cancer. The 1,000 people are represented in the image below. Of these, 10 (1%) DO have colorectal cancer [denoted by the black figures] in the image below. The remaining 990 people (99%) DO NOT have colorectal cancer [denoted by the grey figures].


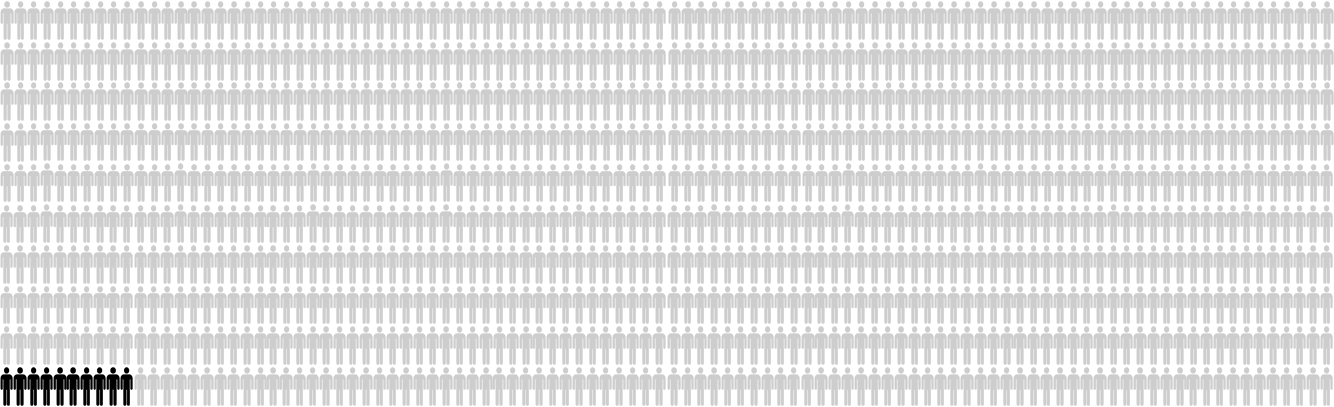


One way of measuring the accuracy of a test for people who DO have colorectal cancer is the **true-positive rate**. The true-positive rate is the number of people *with* *cancer* that are *correctly* *identified* by a *positive* test. For example, looking in more detail at the 10 people [denoted by the black figures] with colorectal cancer from the image above, a true-positive rate of 8 out of 10 (or 80%) can be displayed like this:


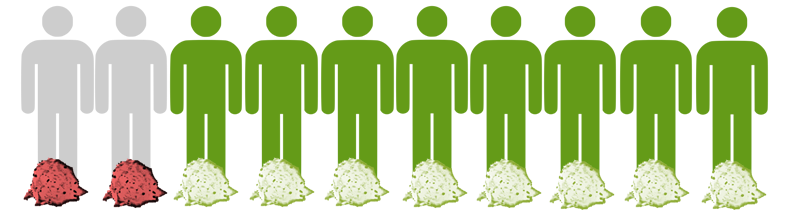


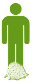
This means that:

8 out of the 10 people (80%) who DO have colorectal cancer are *correctly informed* by the test that they have cancer. This is illustrated by the 8 green people with green cancer cells.


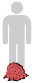
2 out of the 10 people (20%) who DO have colorectal cancer are *incorrectly informed* by the test that they do not have cancer. This is shown by the 2 grey people with red cancer cells.

This means that having more green people with green cancer cells, and less grey people with red cancer cells in the figure describes a test with a higher accuracy for people who DO have colorectal cancer (i.e. higher true positive rate).

**[PROGRAMMER NOTE: DISPLAY THIS QUESTION AFTER 15 SECONDS]**

**Q3. So, looking at the two tests below (i.e. Screening A and Screening B), and assuming the tests are the same except for the true-positive rate, which screening test is better?**

|  |  | **Screening A** |  | **Screening B** |  |
| --- | --- | --- | --- | --- | --- |
| **True-positive rate** (test accuracy if you  DO have cancer) |  | 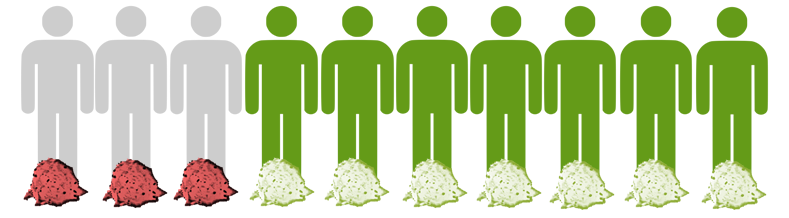  **7 out of 10 (70%)**  individuals *with cancer* will receive a *correct positive result* |  | 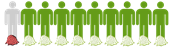  **9 out of 10 (90%)**  individuals *with cancer* will receive a *correct positive result* |  |
| *Which test is better?* |  | 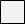 |  | 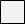 |  |

**[*If Screening A was chosen display:* “This is NOT correct! If ‘Screening A’ is used, only 7 of the 10 people with cancer would be correctly informed that they have cancer, while with ‘Screening B’ 9 of the 10 people with cancer would have been correctly informed that they have cancer. Thus, more cancers are correctly identified, and fewer cancers are missed by ‘Screening B’ than by ‘Screening A’.”
*If Screening B was chosen display: “*Correct! If ‘Screening B’ is used, 9 of the 10 people with cancer would be correctly informed that they have cancer, compared to ‘Screening A’ that would have only correctly informed 7 of the 10 people with cancer that they have cancer. Thus, more cancers are correctly identified, and fewer cancers are missed by ‘Screening B’ than by ‘Screening A’.”]**

In addition to the true-positive rate, the precision of a screening test can also be measured by a **true-negative rate**. Using the same example as before, please imagine that we choose 10 people randomly from the 990 that DO NOT have cancer [denoted by the purple figures:
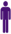
] in the image below.


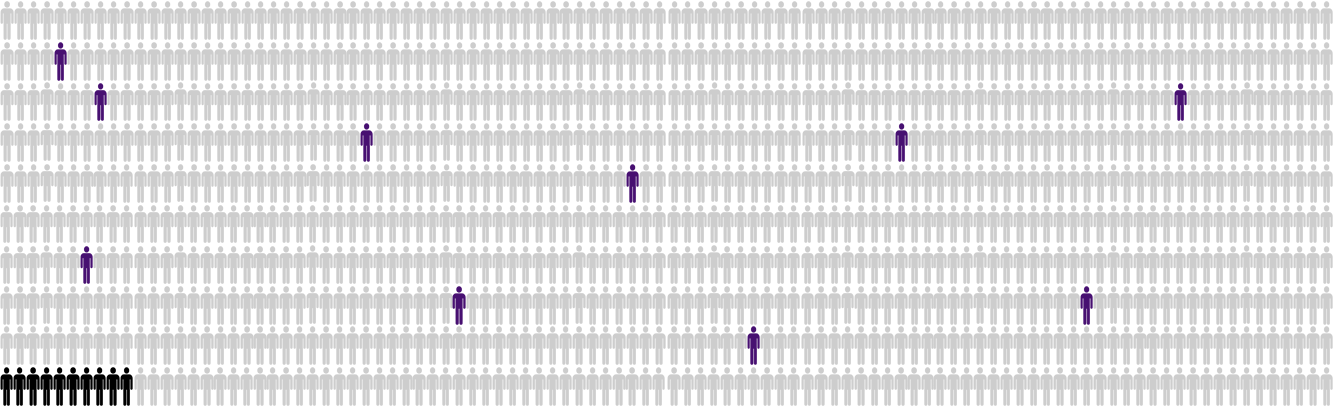


The true-negative rate measures the accuracy of a test for people who DO NOT have colorectal cancer. The true-negative rate is the number of people *without* *cancer* that are *correctly* *identified* by a *negative* test. A true-negative rate of 9 out of 10 (or 90%) can be displayed like this:


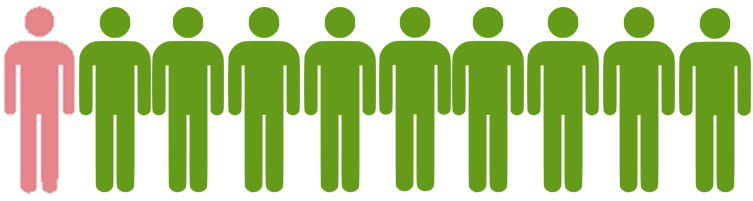


This means that:


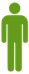
9 out of the 10 people (90%) who DO NOT have colorectal cancer are *correctly informed* by the test that they do not have cancer. This is illustrated by the green figures.


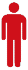
1 out of the 10 people (10%) who DO NOT have colorectal cancer are *incorrectly informed* by the test that they have cancer, despite being cancer free. This means that this one person may undergo unnecessary additional procedures, such as additional colonoscopies. Being incorrectly told they have cancer may also result in unnecessary worry and concerns for individuals, and their families..

This means that having more green people and less red people in the figure, describes a test with a higher accuracy for people who DO NOT have colorectal cancer (i.e. higher true negative rate).

(Please note that while the figure above only displays 10 people that were randomly selected from the 990 people who DO NOT have cancer and underwent screening, if we were to select 10 different people from those who DO NOT have cancer, the result would be the same.)

**Q4. Looking at the two screening tests below (i.e. Screening A and Screening B), and assuming the tests are the same except for the true-negative rate, which one is better?**

|  |  | **Screening A** |  | **Screening B** |  |
| --- | --- | --- | --- | --- | --- |
| **True-negative rate**  (test accuracy if you  DO NOT have cancer) |  | 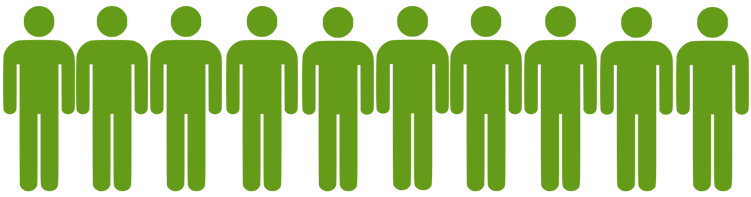  **10 out of 10 (100%)** individuals *without* *cancer* will receive a *correct negative result* |  | 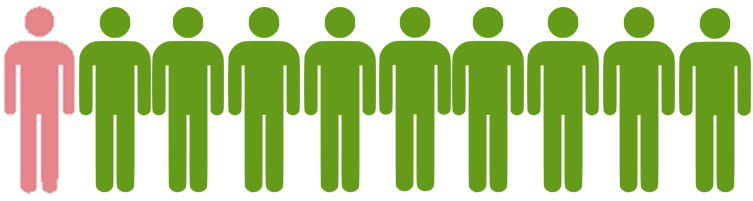  **9 out of 10 (90%)**  individuals *without cancer* will receive a *correct negative result* |  |
| *Which test is better?* |  | 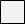 |  | 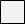 |  |

**[*If Screening A was chosen display:* “Correct! If ‘Screening A’ is used, all 10 cancer free people would be correctly informed that they do not have cancer. However, if ‘Screening B’ is used, 9 of the 10 cancer free people would be correctly informed that they do not have cancer while 1 person would be incorrectly informed that they have cancer. Thus, ‘Screening B’ would place 1 person at risk of undergoing unnecessary procedures such as additional colonoscopies.”**

***If Screening B was chosen display: “*This is NOT correct! If ‘Screening B’ is used, 9 of the 10 cancer free people would be correctly informed that they do not have cancer, while 1 person would be incorrectly informed that they have cancer. Thus, ‘Screening B’ would place 1 person at risk of undergoing unnecessary procedures such as additional colonoscopies. However, if ‘Screening A’ is used, all 10 cancer free people would be correctly informed that they do not have cancer.”]**

**Q5. Please compare the following two screening tests below (i.e. Screening A and Screening B) and select the one correct statement from the options below.**

|  |  | **Screening A** |  | **Screening B** |  |
| --- | --- | --- | --- | --- | --- |
| **True-positive rate**  (test accuracy if you  DO have cancer) |  | 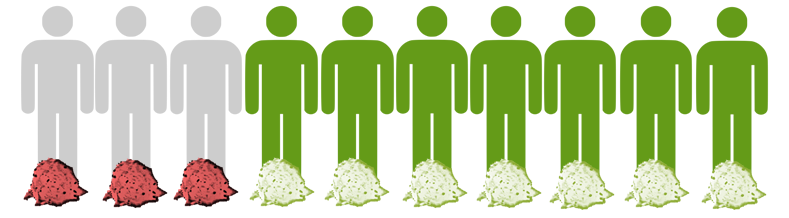  **7 out of 10 (70%)**  individuals *with cancer* will receive a *correct positive result* |  | 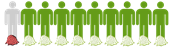  **9 out of 10 (90%)**  individuals *with cancer* will receive a *correct positive result* |  |
| **True-negative rate**  (test accuracy if you  DO NOT have cancer) |  | 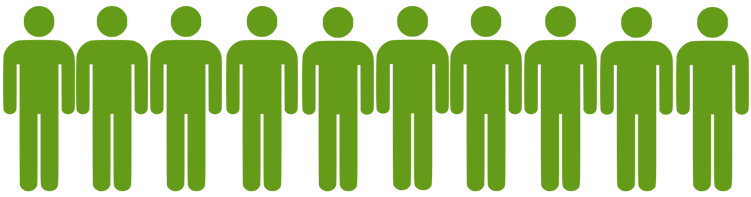  **10 out of 10 (100%)** individuals *without* *cancer* will receive a *correct negative result* |  | 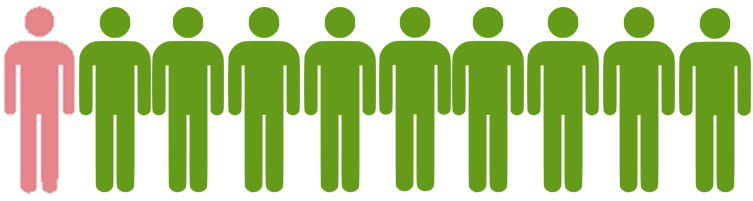  **9 out of 10 (90%)**  individuals *without cancer* will receive a *correct negative result* |  |

❑ Screening A is more likely to find colorectal cancer in people, if it exists.
❑ Screening B is less likely to find people without colorectal cancer that do not have cancer.
❑ Considering the whole sample tested (1,000 people), there are as many people with colorectal cancer as people without colorectal cancer.

**The correct responses are displayed below.**

|  |  | **Screening A** |  | **Screening B** |  |
| --- | --- | --- | --- | --- | --- |
| **True-positive rate**  (test accuracy if you  DO have cancer) |  | 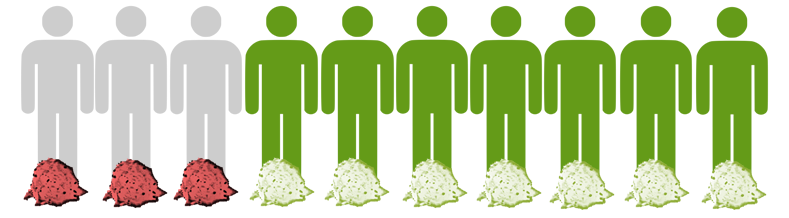  **7 out of 10 (70%)**  individuals *with cancer* will receive a *correct positive result* |  | 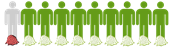  **9 out of 10 (90%)**  individuals *with cancer* will receive a *correct positive result* |  |
| **True-negative rate**  (test accuracy if you  DO NOT have cancer) |  | 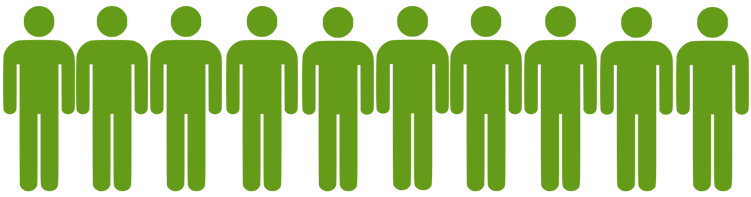  **10 out of 10 (100%)** individuals *without* *cancer* will receive a *correct negative result* |  | 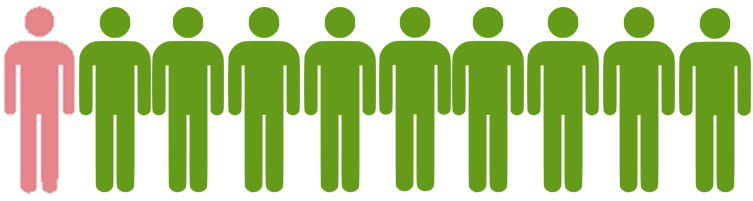  **9 out of 10 (90%)**  individuals *without cancer* will receive a *correct negative result* |  |

**[Select statement 2 (do not allow for selection this is just for feedback purposes)]**
❑ Screening A is more likely to find colorectal cancer in people, if it exists.
***This is NOT correct, because Screening B has a higher true-positive rate than Screening A!***
❑ Screening B is less likely to find people without colorectal cancer that do not have cancer.
***This is correct, because Screening A has a higher true-negative rate than Screening B!***❑ Considering the whole sample tested (the 1,000 people from before), there are as many people with colorectal cancer as people without colorectal cancer.
***This is NOT correct, because in our example of 1,000 people tested for colorectal cancer only a small proportion of 10(1%) HAVE colorectal cancer. The remaining 990 people (99%) DO NOT have colorectal cancer. So, there are NOT as many people with colorectal cancer as people without colorectal cancer.***

Finally, colorectal cancer screening tests can be conducted at different frequencies. The optimal frequency depends on multiple factors, including the accuracy (true positive and true negative rates) of the test or its level of invasiveness.

**Q6. Please compare the following possible frequencies for CRC screening tests and select the option that you would prefer.**

*Please note that screening more regularly may not necessarily increase the chance of finding the cancer.*

| **Every year** | 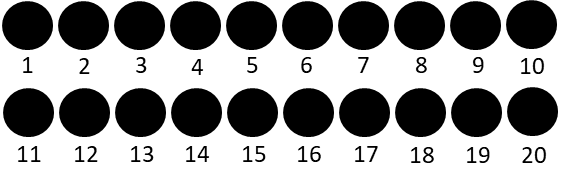 | 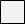 |
| --- | --- | --- |
| **Every 3 years** | 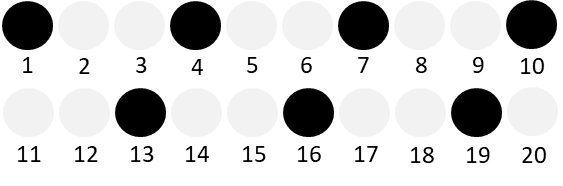 | 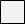 |
| **Every 10 years** | 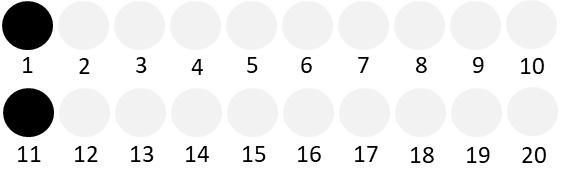 | 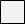 |

| **Section 2: About your Screening Preferences** |
| --- |

In this section you will be asked to make 13 choices between two alternative screening tests (i.e. Screening A and Screening B), represented by different test characteristics for each choice task. **An example of these choice tasks is displayed below.** It is important that you carefully consider and weigh all the different characteristics of the screening options before making a choice. You can hover with your mouse over the screening characteristics in the first columns to remind yourself of what they are.

|  |  | **Screening A** |  | **Screening B** |  |
| --- | --- | --- | --- | --- | --- |
| **Type**  (what type of test  is conducted) |  | 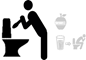  **At-home stool-based test** |  | 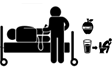**Colonoscopy** |  |
| **Frequency**  (how often the test  is conducted) |  | 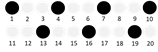  **Every 3 years** |  | 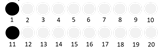**Every 10 years** |  |
| **True-positive rate**  (test accuracy if you  DO have cancer) |  | 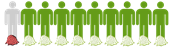 **9 out of 10 (90%)**  individuals *with cancer* will receive a *correct* *positive result* |  | 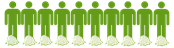 **10 out of 10 (100%)**  individuals *with cancer* will receive a *correct positive result* |  |
| **True-negative rate**  (test accuracy if you  DO NOT have cancer) |  | 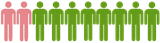 **8 out of 10 (80%)**  individuals *without cancer* will receive a *correct negative result* |  | 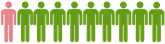 **9 out of 10 (90%)**  individuals *without cancer* will receive a *correct negative result* |  |
|  |  |  |  |  |  |

**Q7. Let’s first try one task for practice:**

|  |  | **Screening A** |  | **Screening B** |  |
| --- | --- | --- | --- | --- | --- |
| **Type**  (what type of test  is conducted) |  | 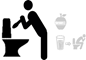  **At-home stool-based test** |  | 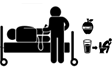**Colonoscopy** |  |
| **Frequency**  (how often the test  is conducted) |  | 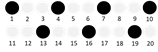  **Every 3 years** |  | 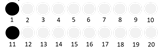**Every 10 years** |  |
| **True-positive rate**  (test accuracy if you  DO have cancer) |  | 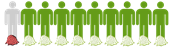 **9 out of 10 (90%)**  individuals *with cancer* will receive a *correct* *positive result* |  | 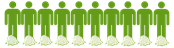 **10 out of 10 (100%)**  individuals *with cancer* will receive a *correct positive result* |  |
| **True-negative rate**  (test accuracy if you  DO NOT have cancer) |  | 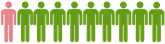 **9 out of 10 (90%)**  individuals *without cancer* will receive a *correct negative result* |  | 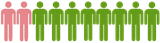 **8 out of 10 (80%)**  individuals *without cancer* will receive a *correct negative result* |  |
|  |  |  |  |  |  |
| *Please make your choice* |  | 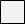 |  | 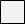 |  |

- **Present DCE according to experimental design**
- **Repeat choice task 5 as seen by respondent**
- **Present dominance test**

**Dominance test**

|  |  | **Screening A** |  | **Screening B** |  |
| --- | --- | --- | --- | --- | --- |
| **Type**  (what type of test  is conducted) |  | **At-home stool-based test**  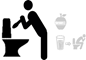 |  | **At-home stool-based test**  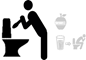 |  |
| **Frequency**  (how often the test  is conducted) |  | **Every 3 years**  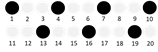 |  | **Every 3 years**  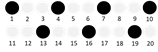 |  |
| **True-positive rate**  (test accuracy if you  DO have cancer) |  | **9 out of 10 (90%)**  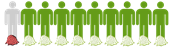 individuals *with cancer* will receive a *correct positive test result* |  | **8 out of 10 (80%)**  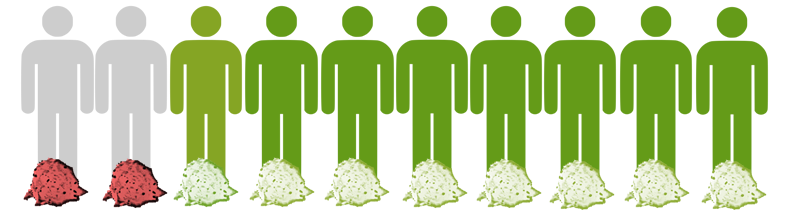 individuals *with cancer* will receive a *correct positive test result* |  |
| **True-negative rate**  (test accuracy if you  DO NOT have cancer) |  | **9 out of 10 (90%)**    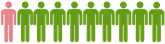 individuals with *no cancer* will  receive a *correct negative test result* |  | **8 out of 10 (80%)**  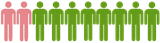 individuals with *no cancer* will receive a *correct negative test result* |  |
|  |  |  |  |  |  |
| *Please make your choice* |  | 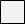 |  | 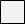 |  |

| **Section 3: General Questions about Colorectal Cancer Testing** |
| --- |

**[Presented if Q17a = yes]**

**Q17c. At what age did you do your first colorectal cancer screening?***______________* years

**[Presented if Q17a = yes]**

**Q17d. How long ago was your most recent colorectal cancer screening test?** *Please select from the dropdown menu below.*

*[0-3 months ago, 3 months – 6 months ago, 6 months – 1 year ago, 1 year – 3 years ago, 3-5 years ago, 5 – 10 years ago, 10 years ago or longer, I do not remember]* **[Presented if Q17a = no; or, if Q17b not colonoscopy]**

**Q18. On a scale from 1 to 10, where 1 is not at all and 10 is extremely, how willing are you to take a colonoscopy once every 10 years?***Please choose a number from the scale below*

| 🔾 | 🔾 | 🔾 | 🔾 | 🔾 | 🔾 | 🔾 | 🔾 | 🔾 | 🔾 |
| --- | --- | --- | --- | --- | --- | --- | --- | --- | --- |
| 1 | 2 | 3 | 4 | 5 | 6 | 7 | 8 | 9 | 10 |
| Not willing at all |  |  |  |  |  |  |  |  | Extremely willing |

**[Presented if Q17a = no; or, if Q17b not blood test]**

**Q18b. On a scale from 1 to 10, where 1 is not at all and 10 is extremely, how willing are you to take a blood test for colorectal cancer screening once every 1 year?***Please choose a number from the scale below*

| 🔾 | 🔾 | 🔾 | 🔾 | 🔾 | 🔾 | 🔾 | 🔾 | 🔾 | 🔾 |
| --- | --- | --- | --- | --- | --- | --- | --- | --- | --- |
| 1 | 2 | 3 | 4 | 5 | 6 | 7 | 8 | 9 | 10 |
| Not willing at all |  |  |  |  |  |  |  |  | Extremely willing |

**[Presented if Q17a = no; or, if Q17b not ‘at-home stool-based test’]**

**Q19. On a scale from 1 to 10, where 1 is not at all and 10 is extremely, how willing are you to take an at-home stool-based test every 1 year?***Please choose a number from the scale below*

| 🔾 | 🔾 | 🔾 | 🔾 | 🔾 | 🔾 | 🔾 | 🔾 | 🔾 | 🔾 |
| --- | --- | --- | --- | --- | --- | --- | --- | --- | --- |
| 1 | 2 | 3 | 4 | 5 | 6 | 7 | 8 | 9 | 10 |
| Not willing at all |  |  |  |  |  |  |  |  | Extremely willing |

**Q19a. On a scale from 1 to 10, where 1 is not at all and 10 is extremely, how willing are you to take an at-home stool-based test every 3 years?***Please choose a number from the scale below*

| 🔾 | 🔾 | 🔾 | 🔾 | 🔾 | 🔾 | 🔾 | 🔾 | 🔾 | 🔾 |
| --- | --- | --- | --- | --- | --- | --- | --- | --- | --- |
| 1 | 2 | 3 | 4 | 5 | 6 | 7 | 8 | 9 | 10 |
| Not willing at all |  |  |  |  |  |  |  |  | Extremely willing |

**Q19b. On a scale from 1 to 10, where 1 is not at all and 10 is extremely, how willing are you to have your kit for a stool-based testing delivered to and picked up from your home, instead of you picking it up from and dropping it back to from your local doctor’s office or healthcare clinic?**

*Please choose a number from the scale below*

| 🔾 | 🔾 | 🔾 | 🔾 | 🔾 | 🔾 | 🔾 | 🔾 | 🔾 | 🔾 |
| --- | --- | --- | --- | --- | --- | --- | --- | --- | --- |
| 1 | 2 | 3 | 4 | 5 | 6 | 7 | 8 | 9 | 10 |
| Not willing at all |  |  |  |  |  |  |  |  | Extremely willing |

**Q19c. On a scale from 1 to 10, where 1 is not at all concerned and 10 is extremely concerned, how concerned are you about colorectal cancer?***Please choose a number from the scale below*

| 🔾 | 🔾 | 🔾 | 🔾 | 🔾 | 🔾 | 🔾 | 🔾 | 🔾 | 🔾 |
| --- | --- | --- | --- | --- | --- | --- | --- | --- | --- |
| 1 | 2 | 3 | 4 | 5 | 6 | 7 | 8 | 9 | 10 |
| Not at all concerned |  |  |  |  |  |  |  |  | Extremely concerned |

**Q20a. Have you ever NOT attended a scheduled colonoscopy?***Please select one answer*

🔾 Yes
🔾 No
🔾 Don’t know

**[Presented if Q20a = yes]**

**Q20b. What were the reasons for you not attending your scheduled colonoscopy?***Please select all that apply*

❑ I forgot
❑ Other commitments or time limitations
❑ Concern about preparation steps
❑ Fear of the procedure
❑ Fear of the findings
❑ Other, please specify ________
🔾 Don’t remember

**Q20c. Have you ever NOT completed a scheduled stool-based test?***Please select one answer*

🔾 Yes
🔾 No
🔾 Don’t know

**[Presented if Q20c = yes]**

**Q20d. What were the reasons for you not to completing your scheduled stool-based test?***Please select all that apply*

❑ I forgot
❑ Other commitments or time limitations
❑ Concern about preparation steps
❑ Fear of the procedure
❑ Fear of the findings
❑ Other, please specify ________
🔾 Don’t remember

**Q21. How much do you agree with each of the following statements below***Please select one answer per row*

|  | Strongly  Agree | Agree | Undecided | Disagree | Strongly Disagree |
| --- | --- | --- | --- | --- | --- |
| I could not make myself take a sample of my own stool | 🔾 | 🔾 | 🔾 | 🔾 | 🔾 |
| I find the idea of taking a sample of my own stool disgusting and/or embarrassing | 🔾 | 🔾 | 🔾 | 🔾 | 🔾 |
| Stool tests are not precise enough for me to use them as a screening test | 🔾 | 🔾 | 🔾 | 🔾 | 🔾 |
| Blood tests are not precise enough for me to use them as a screening test | 🔾 | 🔾 | 🔾 | 🔾 | 🔾 |
| I could not make myself attend a colonoscopy during which a camera is inserted into my rectum | 🔾 | 🔾 | 🔾 | 🔾 | 🔾 |
| Even though colonoscopy is precise, it is also very unpleasant | 🔾 | 🔾 | 🔾 | 🔾 | 🔾 |
| I find the idea of completing a colonoscopy disgusting and/or embarrassing | 🔾 | 🔾 | 🔾 | 🔾 | 🔾 |
| Undergoing a regular colorectal cancer screening is important to me | 🔾 | 🔾 | 🔾 | 🔾 | 🔾 |
| I am afraid of developing cancer | 🔾 | 🔾 | 🔾 | 🔾 | 🔾 |
| Regular colorectal cancer screening could safe my life | 🔾 | 🔾 | 🔾 | 🔾 | 🔾 |
| If I have colorectal cancer, I would prefer not knowing until I have symptoms, even if this reduces my survival chances | 🔾 | 🔾 | 🔾 | 🔾 | 🔾 |
| Colorectal cancer screening must fit into my schedule | 🔾 | 🔾 | 🔾 | 🔾 | 🔾 |
| I am afraid of needles | 🔾 | 🔾 | 🔾 | 🔾 | 🔾 |

| **About you** |
| --- |

**Q22. How would you rate your overall health?***Please choose only one option*

🔾 Very good
🔾 Good
🔾 Fair
🔾 Bad
🔾 Very bad

**Q23a. How often do you have someone help you read medical or healthcare materials?** *Please choose only one option*

| 🔾  Always | 🔾  Often | 🔾  Sometimes | 🔾  Occasionally | 🔾  Never |
| --- | --- | --- | --- | --- |

**Q23b. How confident are you filling out medical forms by yourself?***Please choose only one option*

| 🔾  Extremely | 🔾  Quite a bit | 🔾  Somewhat | 🔾  A little bit | 🔾  Not at all |
| --- | --- | --- | --- | --- |

**Q23c. How often do you have problems learning about your medical condition because of difficulty understanding written information?***Please choose only one option*

| 🔾  Always | 🔾  Often | 🔾  Sometimes | 🔾  Occasionally | 🔾  Never |
| --- | --- | --- | --- | --- |

Medical decisions often include information on probabilities and percentages. The following questions will help us understand how familiar and comfortable you are with this type of information.

**Q24a. Which of the following numbers represents the highest probability/likelihood of getting a disease?***Please choose only one option*

| 🔾  1 in 100 | 🔾  1 in 1000 | 🔾  1 in 10 |
| --- | --- | --- |

**Q24b. Which of the following represents the highest probability/likelihood of getting a disease?***Please choose only one option*

| 🔾  1% | 🔾  10% | 🔾  5% |
| --- | --- | --- |

**Q24c. If the chance of getting a disease is 10%, how many people would be expected to get the disease out of 100? ______ [RANGE 0-9999]**

**Q24d. If the chance of getting a disease is 10%, how many people would be expected to get the disease out of 1000? ______ [RANGE 0-9999]**

**Q24e. If the chance of getting a disease is 20 out of 100, this would be the same as having a ____% chance of getting the disease. [RANGE 0-9999]**

**Q25. What is your age***Please fill in below*

_______________

**Q28. What type of health insurance do you have currently?**

| ❑ | Medicare |
| --- | --- |
| ❑ | Medicaid |
| ❑ | Federal (VA, TRICARE) |
| ❑ | Employer-sponsored/commercial |
| ❑ | Self-purchased commercial insurance |
| ❑ | Uninsured |

**Q29. Which of the following best describes your living situation?***Please select one option*

| 🔾 | Live alone |
| --- | --- |
| 🔾 | Live with partner/spouse and no children |
| 🔾 | Live with partner/spouse and children |
| 🔾 | Live with children |
| 🔾 | Live with family other than partner/spouse or children |
| 🔾 | Live with roommates |
| 🔾 | Live in retirement home |
| 🔾 | Other ______ |

**Q30. What is your highest level of education?***Please select one option*

| 🔾 | Less than high school diploma |
| --- | --- |
| 🔾 | High school diploma |
| 🔾 | Some college |
| 🔾 | College/University degree |
| 🔾 | Postgraduate degree (e.g. Masters, PhD, MBA) |
| 🔾 | Other __________ |

**Q31. What is your current employment status?***Please select all that apply*

| ❑ | Employed Full-time |
| --- | --- |
| ❑ | Employed Part-time |
| ❑ | Stay at home parent or homemaker |
| ❑ | Student |
| ❑ | Not currently employed |
| ❑ | Retired |
| ❑ | Unable to work due to disability |
| ❑ | Other __________ |

**Q32. In which country were you born?***Please select one option from the drop-down menu*

[Insert drop-down menu with list of countries].

**Q33. Which of the following best describes the area in which you currently live?***Please select only one option*

❑ Rural / countryside

❑ Outskirts / suburbs of a small city

❑ Center or close to center of a small city

❑ Outskirts / suburbs of a large city

❑ Center or close to center of a large city

**Q34a. Roughly, how long does it take you to reach your nearest clinic?**

*Please complete below*

____________minutes

**Q34b. Roughly, how long does it take you to reach your nearest hospital?**

*Please complete below*

____________minutes

**Q35a. How convenient it is for you to attend your nearest clinic?**

*Please select only one option*

❑ Very convenient

❑ Convenient

❑ Neither convenient nor inconvenient

❑ Inconvenient

❑ Very inconvenient

**Q35b. How convenient it is for you to attend your nearest hospital?**

*Please select only one option*

❑ Very convenient

❑ Convenient

❑ Neither convenient nor inconvenient

❑ Inconvenient

❑ Very inconvenient

**Q36a. How tall are you?***Please complete below*

**____________** [feet] and ____________ [inches]

**Q36b. What is your weight?**

*Please complete below*

**____________** [pounds (lbs)]

**Q37. Which of the following statements applies to you?***Please select all that apply*

❑ Typically, I eat red meat or processed meat (e.g. beef, pork, sausages) most days a week
❑ Typically, I drink at least one sugary or fizzy drink (e.g. cola, ice tea) every day
❑ Typically, I walk or do other moderate physical activities for fewer than 3 hours a week

❑ Typically, I drink more than one glass of alcohol (e.g. beer, wine, liquor) each day **[f]**

❑ Typically, I drink more than two glasses of alcohol (e.g. beer, wine, liquor) each day **[m]**

❑ Typically, I consume dairy products such as milk, yogurt or cheese every day
❑ I take daily nutrition/food supplements such as vitamin D and/or calcium

❑ I smoke or have smoked for more than 10 years
❑ I took or have been taking oral contraceptives for more than 5 years **[f]**

❑ I have taken or am taking hormones for post-menopausal symptoms relief **[f]**

**Q38. Would you say that you are physically more active, less active, or about as active compared to other persons your age?**

*Please select only one option*

| 🔾 | A lot more |
| --- | --- |
| 🔾 | A little more |
| 🔾 | About the same |
| 🔾 | A little less |
| 🔾 | A lot less |

**Q39. What is your total yearly household income?**

*Please select only one option*

| 🔾 | Less than $25,000 |
| --- | --- |
| 🔾 | $25,000 to $49,999 |
| 🔾 | $50,000 to $74,999 |
| 🔾 | $75,000 to $99,999 |
| 🔾 | $100,000 to $149,999 |
| 🔾 | $150,000 to $200,000 |
| 🔾 | more than $200,000 |
| 🔾 | Prefer not to answer |

**40. How many people live from your total yearly household income?**

*Please select only one option*

| 🔾 | Just me |
| --- | --- |
| 🔾 | Only adults (including me) |
| 🔾 | Children and adults (including me) |
| 🔾 | Prefer not to answer |

**Thank you!**

**Survey: Physicians**

| **Introduction** |
| --- |

Thank you for agreeing to take part in this survey. This study is conducted by the heath research organization Evidera, on behalf of a sponsor to understand physician’s perceptions of and preferences for colorectal cancer screening.

There are four sections in this survey, which will take approximately 30 minutes to complete:

- **Section 1** will review colorectal cancer and colorectal cancer screening modalities to introduce the terminology used in the survey.
- **Section 2** will ask you to make several choices between alternative screening options. This is an established method that helps researchers understand what aspects of screening matter to study participants.
- **Section 3** will ask you some general questions about colorectal cancer screening (e.g. your experience and opinion).
- **Section 4** will ask you about some sociodemographic information about yourself, as well as your clinical experience and setting.

Your responses will be anonymous, and your name will *never* be identified with any of your answers. All data will be used *anonymously* and only used for *medical research* purposes.

We are interested in understanding **your** perspective as physician.

| **Section 1: About Colorectal Cancer Screening** |
| --- |

**About Colorectal Cancer**

- Colorectal cancer is cancer that occurs in the colon (also known as the large bowel or large intestine) or rectum. Sometimes it is also called colon cancer or rectal cancer. The rectum is the passage that connects the colon to the anus. Colorectal cancer is the fourth most common cancer in the U.S. and both men and women have almost equal chances of getting it.

**Illustration of colon and rectum**


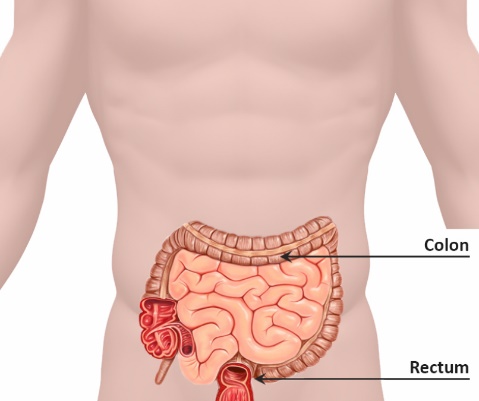


- Colorectal cancer typically starts as adenomas (or polyps) that develop into cancer. Adenomas are lumps grown on the inner lining of the colon or rectum. Only some adenomas will develop into cancer over time. While it is unclear if a polyp will become cancer, adenomas larger than 1cm, or collections of adenomas have an increased risk of becoming cancer. Once a polyp becomes cancer, it grows along and within the walls of the colon or rectum, and eventually spreads through the blood.
- There are several factors that make colorectal cancer more likely to develop. These include a family history of such cancer, being older than 50 years, smoking, being overweight, being physically inactive, consuming a lot of alcohol or certain foods (e.g. regularly eating large amounts of red meat), consuming a low fiber diet (e.g. lack of fruits and vegetables), and having certain types of diseases (e.g. inflammatory bowel diseases).

**About Colorectal Cancer Screening**

Three types of potential screening tests are colonoscopy, at-home stool-based test and blood test:

-
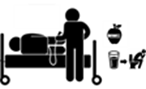
**Colonoscopy:** This test is conducted in a clinic or hospital. A trained physician will insert a thin, flexible tube with a camera into the patient’s rectum to inspect the colon. Patients are usually sedated for this procedure. Individuals will be required to limit their food intake to specific foods (low-fiber) a few days before, and to liquids only the day before the procedure. Preparation also includes drinking a laxative (about half a gallon) the evening before the test, and the morning of the test. The laxative will cause diarrhea to clear the bowels and can in some cases cause dizziness, nausea, or vomiting
- **At-home stool-based test**
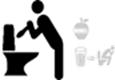
**:** For this test, the patient will be given a test-kit that includes all materials and equipment required for taking a stool sample. The patient will collect the stool in a sample container, which is then sealed in a bag, before returning it for processing. Test-kits may require adding provided chemicals to the stool sample in the sample container. In some cases, test-kits are delivered and picked-up from the patient’s home. In other cases, test-kits need to be picked up from and returned to a local clinic or hospital by the patient. In the case of a positive finding, a follow-up colonoscopy should be undertaken to confirm the finding - i.e. whether colorectal adenoma(s) or cancer is present.
- **
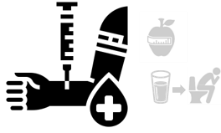
Blood test:** A blood sample is taken by a health care professional in a local clinic or hospital. The health care professional collects a routine sample of blood from the patient by inserting a needle into a vein in the patient’s arm. The blood will then be analysed in a laboratory. In the case of a positive finding, a follow-up colonoscopy should be undertaken to confirm the finding - i.e. whether colorectal adenoma(s) or cancer is present.

**Q1a. In the past 3 months, how many unique patients have you recommended for a colorectal cancer screening [approximately]?**

_______ patients

**[Gastroenterologists only]**
**Q1b. In the past year, how many colonoscopies have you conducted yourself [approximately]?**

_______

**Q2. Thinking of your patients for which you prescribe colorectal cancer screening, approximately what percent get offered the following screening tests as first line of screening?***Please complete percentages to add up to 100%*

____ Fecal Immunochemical Test (FIT)
____ Fecal Occult Blood Test (FOBT)

____ Multi-target Stool DNA Test (mt-sDNA)
____ Colonoscopy

____ CT Colonography (CTC)
____ Flexible Sigmoidoscopy (FS)

____ Blood test for colorectal cancer screening
____ Other _________________

**About the Accuracy of Colorectal Cancer Screening**

Colorectal cancer screening tests differ in type (e.g. colonoscopy vs at-home stool-based test), but also in **accuracy.** For example, the accuracy of colonoscopy may depend on the person conducting the test (e.g. experience), how well people followed the preparation steps (e.g. drinking all the laxative, following the diet) and the condition of the bowel.


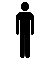
Please imagine that 1,000 people are screened for colorectal cancer. The 1,000 people are represented in the image below. Of these, 10 (1%) DO have colorectal cancer [denoted by the black figures] in the image below. The remaining 990 people (99%) DO NOT have colorectal cancer [denoted by the grey figures].


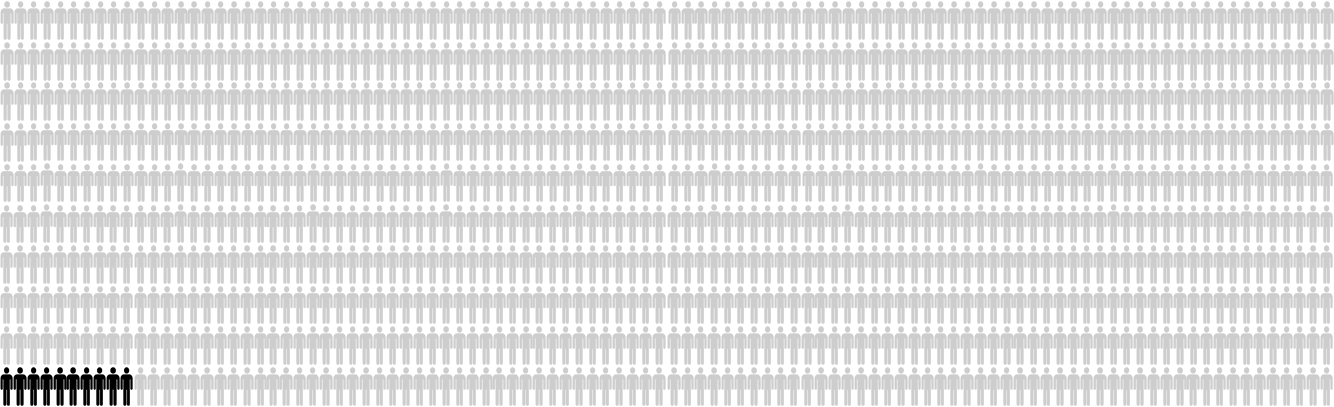


One way of measuring the accuracy of a test for people who DO have colorectal cancer is the **true-positive rate**. The true-positive rate is the number of people *with* *cancer* that are *correctly* *identified* by a *positive* test. For example, looking in more detail at the 10 people with colorectal cancer [denoted by the black figures] from the image above, a true-positive rate of 8 out of 10 (or 80%) can be displayed like this:


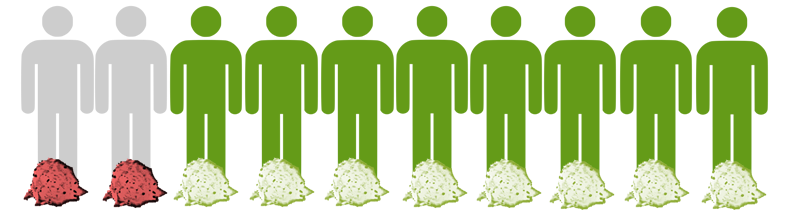


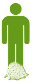
This means that:

8 out of the 10 people (80%) who DO have colorectal cancer are *correctly informed* by the test that they have cancer. This is illustrated by the 8 green people with green cancer cells.


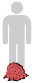
2 out of the 10 people (20%) who DO have colorectal cancer are *incorrectly informed* by the test that they do not have cancer. This is shown by the 2 grey people with red cancer cells. The number of grey people with red cancer cells can also be referred to as the false-negative rate.

This means that having more green people with green cancer cells, and less grey people with red cancer cells in the figure, describes a test with a higher accuracy for people who DO have colorectal cancer (i.e. higher true positive rate).

**Q3. So, looking at the two tests below (i.e. Screening A and Screening B), and assuming the tests are the same except for the true-positive rate, which screening test is better?**

|  |  | **Screening A** |  | **Screening B** |  |
| --- | --- | --- | --- | --- | --- |
| **True-positive**  (test accuracy when the patient DOES have cancer) |  | 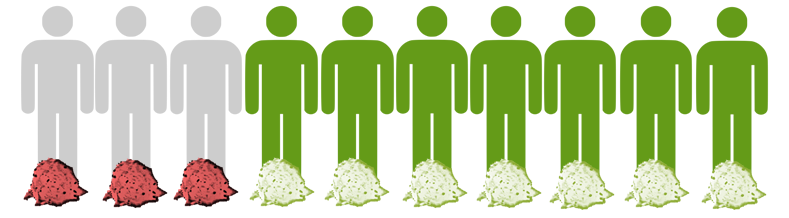  **7 out of 10 (70%)**  individuals *with cancer* will receive a *correct positive result* |  | 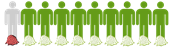  **9 out of 10 (90%)**  individuals *with cancer* will receive a *correct positive result* |  |
| *Which test is better?* |  | 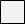 |  | 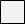 |  |

**[*If Screening A was chosen display:* “This is NOT correct! If ‘Screening A’ is used, only 7 of the 10 people with cancer would be correctly informed that they have cancer, while if ‘Screening B’ is used, 9 of the 10 people with cancer would have been correctly informed that they have cancer. Thus, more cancers are correctly identified, and fewer cancers are missed by ‘Screening B’ than by ‘Screening A’.”
*If Screening B was chosen display: “*Correct! If ‘Screening B’ is used, 9 of the 10 people with cancer would be correctly informed that they have cancer, compared to ‘Screening A’ that would have only correctly informed 7 of the 10 people with cancer that they have cancer. Thus, more cancers are correctly identified, and fewer cancers are missed by ‘Screening B’ than by ‘Screening A’.”]**

In addition to the true-positive rate, the precision of a screening test can also be measured by a **true-negative rate**. Using the same example as before, please imagine that we choose 10 people randomly from the 990 that *do not* have cancer [denoted by the purple figures in the image below].


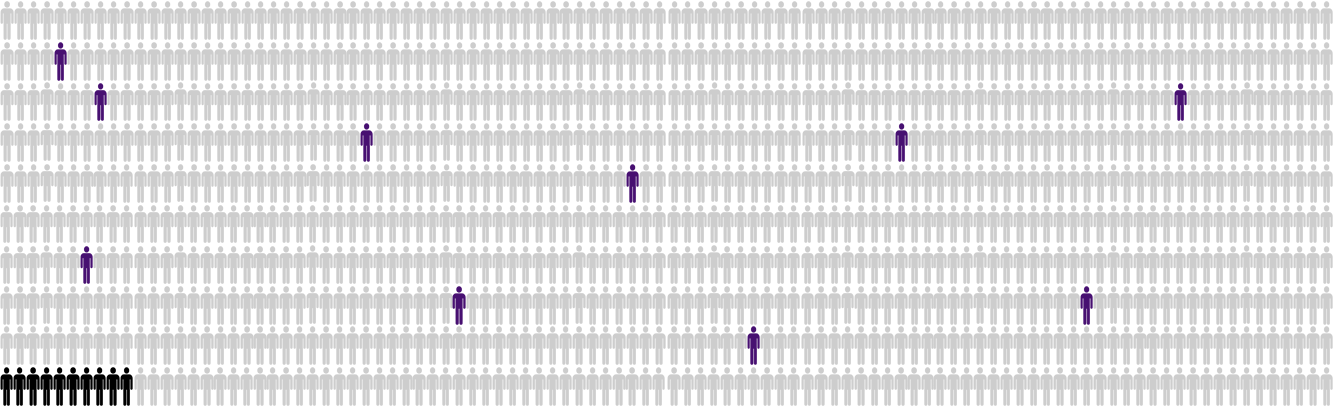


The true-negative rate measures the accuracy of a test for people who DO NOT have colorectal cancer. The true-negative rate is the number of people *without* *cancer* that are *correctly* *identified* by a *negative* test. A true-negative rate of 9 out of 10 (or 90%) can be displayed like this:


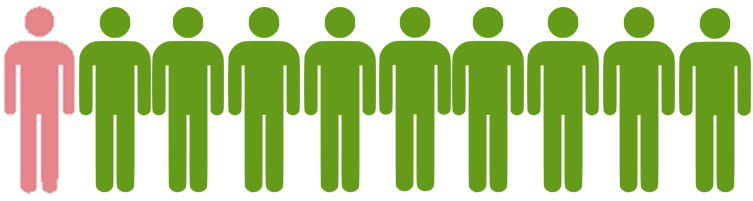


This means that:


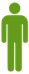
9 out of the 10 people (90%) who DO NOT have colorectal cancer are *correctly informed* by the test that they do not have cancer. This is illustrated by the green figures.


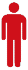
1 out of the 10 people (10%) who DO NOT have colorectal cancer are *incorrectly informed* by the test that they have cancer, despite being cancer free. This means that this one person may undergo unnecessary additional procedures such as additional colonoscopies. Being incorrectly told they have cancer may also result in unnecessary worry and concerns for patients and their families. The number of red people can also be referred to as the false-positive rate.

This means that having more green people and less red people in the figure describes a test with a higher accuracy for people who DO NOT have colorectal cancer (i.e. higher true negative rate).

**Q4. Looking at the two screening tests below (i.e. Screening A and Screening B), and assuming the tests are the same except for the true-negative rate, which one is better?**

|  |  | **Screening A** |  | **Screening B** |  |
| --- | --- | --- | --- | --- | --- |
| **True-negative**  (test accuracy when the patient DOES NOT have cancer) |  | 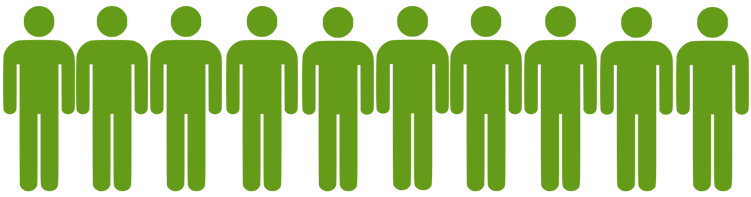  **10 out of 10 (100%)** individuals *without* *cancer* will receive a *correct negative result* |  | 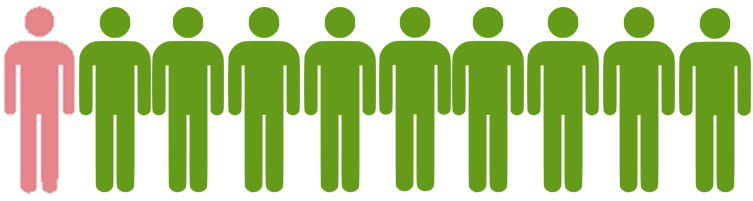  **9 out of 10 (90%)**  individuals *without cancer* will receive a *correct negative result* |  |
| *Which test is better?* |  | 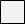 |  | 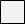 |  |

**[*If Screening A was chosen display:* “Correct! If ‘Screening A’ is used, all 10 cancer free people would be correctly informed that they do not have cancer. However, if ‘Screening B’ is used, 9 of the 10 cancer free people would be correctly informed that they do not have cancer while 1 person would be incorrectly informed that they have cancer.”
*If Screening B was chosen display: “*This is NOT correct! If ‘Screening B’ is used, 9 of the 10 cancer free people would be correctly informed that they do not have cancer while 1 person would be incorrectly informed that they have cancer. However, if ‘Screening A’ is used, all 10 cancer free people would be correctly informed that they do not have cancer.”]**

Screening tests such as colonoscopy and stool tests can detect individuals with advanced adenomas that are at elevated risk of becoming malignant (>1 cm in size). The true-positive rate for detecting individuals with advanced adenomas can be displayed as


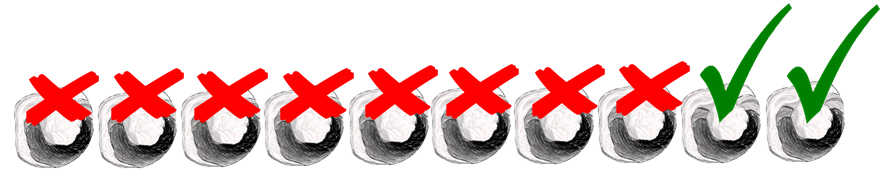


This means that:


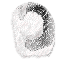

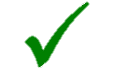


2 out of 10 individuals with advanced adenomas (20%) at elevated risk of becoming malignant (>1 cm in size) are *correctly* detected by the test


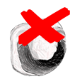
8 out of 10 individuals with advanced adenomas (80%) at elevated risk of becoming malignant (>1 cm in size) are *missed* by the test

**Q5. Looking at the two tests below, and assuming the tests are the same except for the true-positive rate for adenomas, which one is better?**

|  |  | **Screening A** |  | **Screening B** |  |
| --- | --- | --- | --- | --- | --- |
| **Adenoma  true-positive rate**  (test accuracy when the patient DOES have advanced adenomas) |  | 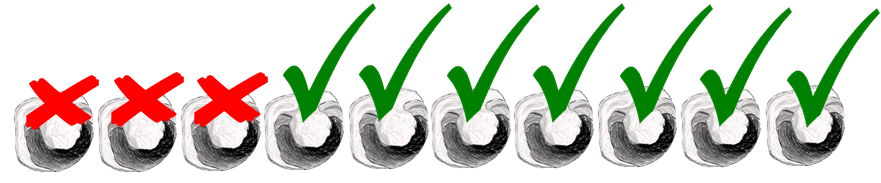  **7 out of 10 (70%)** individuals with advanced adenomas at risk of developing into cancer are correctly identified |  | 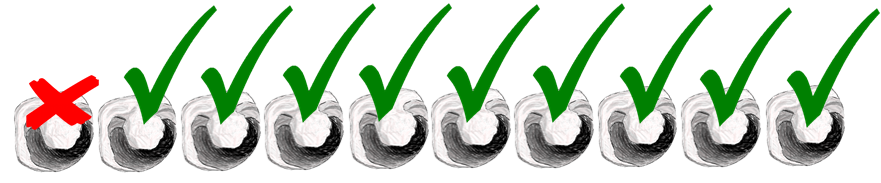 **9 out of 10 (90%)**  individuals with advanced adenomas at risk of developing into cancer are correctly identified |  |
| *Which test is better?* |  | 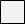 |  | 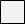 |  |

**[*If Screening A was chosen display:* “This is NOT correct! While ‘Screening A’ identifies 7 of the 10 individuals with advanced adenomas, ‘Screening B’ identifies 9 of the 10 individuals with advanced adenomas. Thus, ‘Screening B’ misses fewer individuals with advanced adenomas than ‘Screening A’.”
*If Screening B was chosen display: “*Correct! While ‘Screening A’ identifies 7 of the 10 individuals with advanced adenomas, ‘Screening B’ identifies 9 of the 10 individuals with advanced adenomas. Thus, ‘Screening B’ misses fewer individuals with advanced adenomas than ‘Screening A’.”]**

Finally, colorectal cancer screening tests can be conducted at different frequencies. The optimal frequency depends on multiple factors, including the accuracy of the test (true positive and true negative rates) or its level of invasiveness.

Below are three possible frequency options for different CRC screening tests.

| **Every year** | 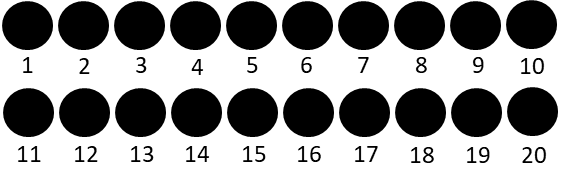 |  |
| --- | --- | --- |
| **Every 3 years** | 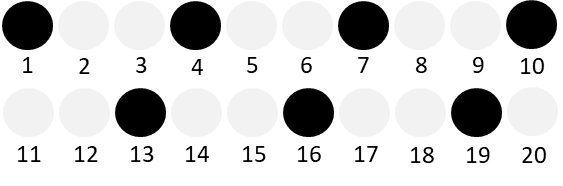 |  |
| **Every 10 years** | 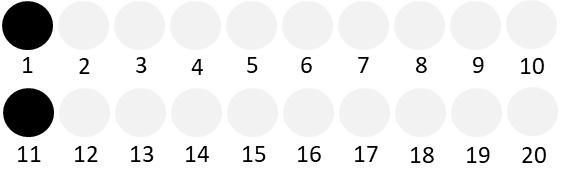 |  |

| **Section 2: About your Screening Preferences** |
| --- |

In this section you will be asked to make 15 choices between two alternative screening tests (i.e. Screening A and Screening B), represented by different test characteristics for each choice task. **An example of these choice tasks is displayed below.** It is important that you carefully consider and weigh all the different characteristics of the screening options before making a choice. You can hover with your mouse over the screening characteristics in the first columns to remind yourself of the definition.

|  |  | **Screening A** |  | **Screening B** |  |
| --- | --- | --- | --- | --- | --- |
| **Type**  (what type of test is conducted) |  | 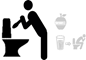  **At-home stool-based test** |  | 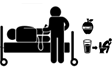 **Colonoscopy** |  |
| **Frequency**  (how often the test is conducted |  | 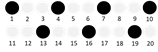  **Every 3 years** |  | 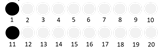**Every 10 years** |  |
| **True-positive**  (test accuracy when the patient DOES have cancer) |  | 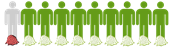  **9 out of 10 (90%)**  individuals *with cancer* will receive a *correct positive result* |  | **10 out of 10 (100%)**  individuals *with cancer* will receive a *correct positive result* |  |
| **True-negative**  (test accuracy when the patient DOES NOT have cancer) |  | **9 out of 10 (90%)** individuals *without cancer* will receive a *correct negative result* |  | **8 out of 10 (80%)**  individuals *without cancer* will receive a *correct negative result* |  |
| **Adenoma  true-positive rate**  (test accuracy when the patient DOES have advanced adenomas) |  | **2 out of 10 (20%)**  individuals with advanced adenomas at risk of developing into cancer are correctly identified |  | **5 out of 10 (50%)**  individuals with advanced adenomas at risk of developing into cancer are correctly identified |  |

**Let’s first work with one task as practice:**

|  |  | **Screening A** |  | **Screening B** |  |
| --- | --- | --- | --- | --- | --- |
| **Type**  (what type of test is conducted) |  | **At-home stool-based test** |  | **Colonoscopy** |  |
| **Frequency**  (how often the test is conducted |  | **Every 3 years** |  | **Every 10 years** |  |
| **True-positive**  (test accuracy when the patient DOES have cancer) |  | **9 out of 10 (90%)**  individuals *with cancer* will receive a *correct positive result* |  | **10 out of 10 (100%)**  individuals *with cancer* will receive a *correct positive result* |  |
| **True-negative**  (test accuracy when the patient DOES NOT have cancer) |  | **9 out of 10 (90%)** individuals *without cancer* will receive a *correct negative result* |  | **8 out of 10 (80%)**  individuals *without cancer* will receive a *correct negative result* |  |
| **Adenoma  true-positive rate**  (test accuracy when the patient DOES have advanced adenomas) |  | **2 out of 10 (20%)**  individuals with advanced adenomas at risk of developing into cancer are correctly identified |  | **5 out of 10 (50%)**  individuals with advanced adenomas at risk of developing into cancer are correctly identified |  |
|  |  |  |  |  |  |
| *Please make your choice* |  |  |  |  |  |
|  |  |  |  |  |  |

- **Present DCE according to experimental design**
- **Repeat choice task 5 as seen by respondent**
- **Present dominance test**

**Dominance test**

|  |  | **Screening A** |  | **Screening B** |  |
| --- | --- | --- | --- | --- | --- |
| **Type**  (what type of test is conducted) |  | **At-home stool-based test** |  | **At-home stool-based test** |  |
| **Frequency**  (how often the test is conducted |  | **Every 3 years** |  | **Every 3 years** |  |
| **True-positive**  (test accuracy when the patient DOES have cancer) |  | **9 out of 10 (90%)**  individuals *with cancer* will receive a *correct positive result* |  | **8 out of 10 (80%)**  individuals *with cancer* will receive a *correct positive result* |  |
| **True-negative**  (test accuracy when the patient DOES NOT have cancer) |  | **9 out of 10 (90%)** individuals *without cancer* will receive a *correct negative result* |  | **8 out of 10 (80%)** individuals *without cancer* will receive a *correct negative result* |  |
| **Adenoma  true-positive rate**  (test accuracy when the patient DOES have advanced adenomas) |  | **5 out of 10 (50%)** individuals with advanced adenomas at risk of developing into cancer are correctly identified |  | **2 out of 10 (20%)**  individuals with advanced adenomas at risk of developing into cancer are correctly identified |  |
|  |  |  |  |  |  |
| *Please make your choice* |  |  |  |  |  |

| **Section 3: General Questions about Colorectal Cancer Testing** |
| --- |

**Q21. On average, how satisfied are you with the available options for colorectal cancer screening (excluding colonoscopy)?**  *Please select one number from the scale below*

| 1 = Totally *unsatisfied* | | | | |  |  |  | 7 = Totally *satisfied* | |
| --- | --- | --- | --- | --- | --- | --- | --- | --- | --- |
|  | 1 | 2 | 3 | 4 | 5 | 6 | 7 |  |  |
|  | ❑ | ❑ | ❑ | ❑ | ❑ | ❑ | ❑ |  |  |

**Q22. To what extent do you perceive that there is a need for improvement in the following aspects of colorectal cancer screening?***Please select only one option per item*

|  |  | **1= No  improvement needed** | | |  | **7 = Major improvement needed** | | |
| --- | --- | --- | --- | --- | --- | --- | --- | --- |
|  |  | **1** | **2** | **3** | **4** | **5** | **6** | **7** |
| **Burden for patients** | | 🔾 | 🔾 | 🔾 | 🔾 | 🔾 | 🔾 | 🔾 |
| **Patient willingness to screen** | | 🔾 | 🔾 | 🔾 | 🔾 | 🔾 | 🔾 | 🔾 |
| **Cost/coverage of screening** | | 🔾 | 🔾 | 🔾 | 🔾 | 🔾 | 🔾 | 🔾 |
| **Patients awareness of screening** | | 🔾 | 🔾 | 🔾 | 🔾 | 🔾 | 🔾 | 🔾 |
| **The precision of screening tests** | | 🔾 | 🔾 | 🔾 | 🔾 | 🔾 | 🔾 | 🔾 |
| **Available capacity for colonoscopy** | | 🔾 | 🔾 | 🔾 | 🔾 | 🔾 | 🔾 | 🔾 |
| **Screening earlier than the age of 50** | | 🔾 | 🔾 | 🔾 | 🔾 | 🔾 | 🔾 | 🔾 |

**Q23. At what age would you recommend starting colorectal cancer screening for a person at average risk?
_________**

| **Section 4: About you** |
| --- |

**Q24. What is your age?***Please fill in below*

_____________

**Q25. How do you identify your gender?***Please select one option*

| 🔾 | Female |
| --- | --- |
| 🔾 | Male |
| 🔾 | Transgender Female |
| 🔾 | Transgender Male |
| 🔾 | Non-binary/Non-conforming |
| 🔾 | Other ______________ |

**Q26a. How long have you been practicing medicine?***Please complete the box below*

__________ years

**[For primary care physicians]**

**Q26b. What is your primary care specialization?***Please select one option*

| 🔾 | Family medicine |
| --- | --- |
| 🔾 | Internal medicine |

**Q27a. How long have you been recommending colorectal cancer screening tests?***Please complete the field below*

__________ years

**[For GI physicians]**

**Q27b. How long have you been conducting colonoscopies?***Please select one option*

| 🔾 | <1 year |
| --- | --- |
| 🔾 | 1-5 years |
| 🔾 | 5-10 years |
| 🔾 | >10 years |
| 🔾 | I have never conducted a colonoscopy myself |

**Q28. What type of clinic/facility do you work in?***Please select only one option*

| 🔾 | Multi-specialty group |
| --- | --- |
| 🔾 | Single specialty group |
| 🔾 | Solo practice |
| 🔾 | Academic system or hospital |
| 🔾 | Other_______________ |

**Q29. Which of the following best describes the location of your primary practice setting?***Please select only one option*

| 🔾 | Rural / countryside |
| --- | --- |
| 🔾 | Outskirts / suburbs of a small city |
| 🔾 | Center or close to center of a small city |
| 🔾 | Outskirts / suburbs of a large city |
| 🔾 | Center or close to center of a large city |

**Thank you!**

## Supplemental file 2: Quantitative Pilot

The quantitative pilot, in combination with the insights from the qualitative pilot, aimed to confirm a design that carefully balanced participants’ cognitive burden while maximizing statistical efficiency. The quantitative pilot of the survey recruited a total of 200 members of the US population at average risk for CRC, 50 PCPs and 50 gastroenterologists (total sample N=300). As in the qualitative phase, participants received access to the survey through a weblink, which included the electronic consent, screening questionnaire, and the link to the full survey for piloting. Participants were expected to complete the survey in approximately 30 minutes. Following completion, they would be remunerated for their time according to their standing agreements with their online access panels. The DCE results were analyzed as described in the Methods.

After the first 100 members (50%) had completed their surveys, a preliminary analysis of the data was completed to confirm that changes made based on the qualitative pilot were providing precise results.

**Results**

A sample of N=202 (47.5% males) members of the US general population at average risk for CRC and N=100 physicians completed the quantitative pilot. The sample of IAR had a mean age of 58.1 years (SD 8.9, range: 45 to 75) and the physician sample had a mean age of 53 years (SD 11.2, range: 31 to 72). Data from this quantitative pilot were carried over to the main study.

The results of the quantitative pilot confirmed discrimination between attribute levels and provided initial insights into participants preferences and how these can differ between the general population and physicians. The model at this stage already identified highly significant (i.e. p<0.001) preferences for any improvements in true-positive and true-negative rates, and for improvements in adenoma true-positive rate in the case of the physicians. In both samples, true-positive rate was the attribute with the biggest influence on participant preferences (IAR marginal utilities, 0.592-2.045; physician marginal utilities, 1.143-2.549; p<0.001) followed closely by adenoma true-positive rate in the case of the physicians sample (marginal utilities, 0.867-2.442; p<0.001). A relevant difference between sample groups (general population and physicians) identified at this stage was the preference for type of test; whereas physicians seemed to prefer colonoscopy over the at-home stool-based test (-0.444; p<0.01), IAR appeared to prefer either blood test (0.276; p<0.01) or at-home stool-based test (0.455; p<0.001) over colonoscopy. No interaction effects were identified, so testing for interactions was suppressed from the experimental design from this point forward.

## Supplemental file 3: Qualitative Pilot Methodology

A qualitative pilot study was completed to pre-test the survey. This qualitative pilot aimed to explore if the chosen attributes, their levels and the format of their presentation were complete, meaningful to participants and understood. For this purpose, six participants from the individuals at average risk for CRC in US, three primary care physicians (PCPs) and three gastroenterologists (total sample N=12) were recruited to participate in one-to-one semi-structured interviews while completing the online survey.

The qualitative pilot was performed as one-on-one computer-assisted phone interviews, with a shared screen or linked online survey format, allowing the interviewer and the participant to follow the survey completion at the same time. These interviews were voice-recorded, and with notes taken by the interviewer. Three main objectives were addressed with this qualitative pilot:

1. To assess if the chosen DCE attributes and levels are relevant, tradeable, and understandable by participants
2. To assess if all attribute levels cover the relevant valuation ranges
3. To assess complexity, clarity, and completeness of the online questionnaire

Within the DCE section of the survey, each attribute was assessed to see if it is tradeable and understood by participants. A special focus was placed on participants’ understanding of true-positive and true-negative, so they were asked to explain in their own words the meaning of these two concepts.

Each interview lasted for approximately 60 minutes and upon completion of the interviews, participants were remunerated with $60 for the general population and $225 to $275 for physicians.

The analysis of the qualitative pilot was based on pre-specified structured notes completed by the interviewer in real time while completing each interview.

**Results**

A sample of 6 members of the US general population in average risk for CRC and 6 physicians (3 primary care physicians and 3 gastroenterologists) completed their interviews. The average age of the general population sample was 52 years, with a minimum age of 45 years and a maximum age of 66 years. Based on the interview findings, the changes in the table below were recommended and implemented.

**Qualitative Pilot: Recommended Changes**

| Section/Question | Suggested Action | Comments |
| --- | --- | --- |
| Introduction | Substitute word "rectum" for "rectal" | change implemented |
| Q1b | Expand the limit of digits up to 4 | change implemented |
| Q2 | As some participants struggled with the technical names of the tests, we suggested reducing technical names to just the common name. For example, High-sensitivity Guaiac-based Fecal Occult Blood Test (HSgFOBT) changed to just Fecal Occult Blood Test (FOBT) | change implemented |
| Q2 | Add blood test to list of options | change implemented |
| Q3 to Q5 (True-positive and True-negative) | This section was restructured to include the following changes:  - represent images within the text - providing a better link between images and text  - rephrase the meaning of True-Positive and True-Negative, using feedback from "in your own words" descriptions  - present attributes on the DCE with a brief description underneath  - separate images and text on the DCE  - after Q5, conclude with one line summarizing the differences between T-N and T-P.  -FREQUENCIES: clarify "Please note that screening more regularly may not necessarily increase the chance of finding the cancer." | changes implemented and all reflected protocol version 2.0 |
| DCE | a) Re-word instructions on example to clarify that it is an example and that participants are comparing columns and not rows (same when comparing true-positive and true-negative on Q3 to Q5)  b) add definitions of attributes below the title (image on side)  c) delete Opt-Out option as this was never chosen (no relevance)  d) minor changes to the experimental design | changes implemented and all reflected protocol version 2.0 |

## Supplemental file 4: Statistical Methods

**Mixed Logit (MXL) Model**

The DCE data was analyzed within the random utility maximization (RUM) framework. This framework represented preferences by a utility function ($u_{ntj}$) that was split into a deterministic component ($v_{ntj}$) and an extreme value distributed error ($\varepsilon_{ntj}$):

$$u_{ntj}=v_{ntj}+\varepsilon_{ntj}$$

The deterministic component was defined as a linear dummy-coded function of the attributes:

$v(x_{jnt})= \alpha+\beta_{1}\mathrm{stool}_{jnt}+\beta_{2}\mathrm{blood}_{jnt}+\beta_{3}{every.3yrs}_{jnt} +\beta_{4}{every.10yrs}_{jnt}+\beta_{5}{TP.70\%}_{jnt}+\beta_{6}{TP.80\%}_{jnt}+$ $\beta_{7}{TP.90\%}_{jnt}+\beta_{8}{TP.100\%}_{jnt}+\beta_{9}{TN.80\%}_{jnt}+\beta_{10}{TN.90\%}_{jnt}+{\beta_{11}TN.100\%}_{jnt}+\beta_{12}{adenoma.TP.50\%}_{jnt}+{\beta_{13}adenoma.TP.100\%}_{jnt}$

The $\beta$ parameters were considered to be individual specific marginal utilities. Thus, the MXL assumed *preference heterogeneity* in the population. Each marginal utility $\beta_{kn}$ followed a normal distribution. The mean ($\bar{\beta}_{k}$) and SD ($\sigma_{k}$) of each distribution were estimated. This was implemented by defining each marginal utility as

| $\beta_{kn}=\bar{\beta}_{k}+\sigma_{k}\xi_{kn}$ |  |
| --- | --- |

where $\xi_{kn}$ was an individual and marginal utility specific draw from the underlying probability distribution.

**Relative Attribute Importance (RAI) Scores**

The difference in marginal utilities between the best level and the worst level of the same attribute provides an estimate of the relative importance of that attribute over the range of levels included in the DCE:

$$\mathrm{RAI}_{k}=100\times\frac{\hat{\beta}_{k}^{\max}-\hat{\beta}_{k}^{\min}}{\sum_{k\in[1;K]} \left( \hat{\beta}_{k}^{\max}-\hat{\beta}_{k}^{\min} \right)}$$

where ($\hat{\beta}_{k}^{\max}-\hat{\beta}_{k}^{\min}$) corresponds to the largest change in utility obtained with moving from the worst (least desirable) to best (most desirable) level of the kth attribute. A bootstrapping procedure with 1000 iterations was implemented to compute the 95% confidence intervals.

**Predicted Choice Probabilities**

Predicted choice probabilities were the logit probabilities with the estimated marginal utilities and the specified attribute levels representing the screening alternatives.

**Average Partial Effects**

Average partial effects were the average change in logit choice probabilities resulting from each attribute level change over the full factorial combination of attributes and levels.

**Supplemental file 5: Attitudes to CRC screening among individuals at average risk (IAR)**

Screening attitudes were assessed in IAR to determine if they had heard of CRC screening types and how they viewed the severity of CRC and the need to utilize screenings. Most IAR had heard of CRC screening (n=1,085; 86.9%), and the most recognized screening types were colonoscopy (n=1,024; 82.0%) and at-home stool-based tests (n=837; 67.0%). Over half (n=723; 57.9%) had previously undergone CRC screening and the mean age at first CRC screening was 50.7 ± 7.9 years. Most IAR (n=992; 79.4%) were concerned about developing colorectal cancer. In general, patients viewed CRC screening as lifesaving and important, although 60.8% of patients believed colonoscopy to be unpleasant and embarrassing. A minority of patients considered stool tests (14.3%) and blood tests (10.6%) to be imprecise at cancer detection.

## Supplemental file 6: Characteristics of physicians’ private practices and experience

Most physicians practiced in a single-specialty (n=175; 43.8%) or multiple-specialty group (n=107; 26.8%) and the median duration of practicing medicine was 22 years. Physicians had recommended CRC screening tests for a median time of 21.5 years ± 9.4 years. Clinical practices were primarily located in the outskirts of large cities (n=126; 31.5%) or within a large city (n=106; 26.5%).

As part of the online survey, physicians were asked to rate the perceived need for improving the seven CRC screening aspects on a scale from 1 to 7, where 1 meant “no improvement needed” and 7 meant “major improvement needed.” The three areas of CRC screening for which physicians perceived the highest need for improvements were:

- Patients’ willingness to screen: 52.0% of physicians reported a high (6 to 7) need for working on improving the willingness to screen among IAR. In line with physicians’ concerns, 38.6% of IAR in the survey did not agree or strongly agree with CRC screening being important to them.
- Screening earlier than the age of 50: 49.5% of physicians reported a high (6 to 7) need of starting CRC screening earlier. When being asked at what age CRC screening should start, physicians reported an average age of 45.6 years (SD 3.8), which is in line with guidance from the American Cancer Society, but younger than the recommended 50 years from the USPSTF.
- The precision of CRC screening: 45.2% physicians reported a high (6 to 7) need for improving the precision of CRC screening.

# Supplemental figures

**Supplemental figure 1. Study overview.**

**Supplemental figure 2. Disposition of individuals at average risk who completed the discrete choice experiment.** Numbers include participants of the quantitative pilot survey. Abbreviation: CRC, colorectal cancer.

**Supplemental figure 3. Colorectal cancer (CRC) screening concepts among individuals at average risk (IAR).** IAR were asked to complete an attitudinal questionnaire where they denoted their level of agreement with statements about CRC and CRC screening on a 5-point agreement scale ranging from 1 (strongly agree) to 5 (strongly disagree). Additionally, a set of questions rated on a 10-point analog scale was implemented to report participants’ willingness (0= not willing at all and 10= extremely willing) to screen if they had not been screened before by colonoscopy, home-based stool test, and/or blood-test.

**Supplemental figure 4. Disposition of physicians who completed the discrete choice experiment.** Abbreviations: CRC, colorectal cancer; GP, general practitioner.

**Supplemental figure 5. Perceived need for colorectal cancer screening improvement among physicians.** Physicians ranked items on a scale from 1-7 where 1 meant “no improvement needed” and 7 meant “major improvement needed”.

**A)**

**B)**

**Supplemental figure 6. Relative importance of attributes among individuals at average risk (A) and physicians (B).** Whiskers denote corresponding 95% confidence interval.

**Supplemental figure 7. Screening test preferences of individuals at average risk by age group.** Whiskers denote corresponding 95% confidence interval. Abbreviations: CRC, colorectal cancer; FIT, fecal immunochemical test; mt-sDNA, multitarget stool DNA

**Supplemental figure 8. Screening test preferences of individuals at average risk by race.** Whiskers denote corresponding 95% confidence interval. Abbreviations: CRC, colorectal cancer; FIT, fecal immunochemical test; mt-sDNA, multitarget stool DNA

**Supplemental figure 9. Screening test preferences for physicians by specialization.** Whiskers denote corresponding 95% confidence interval. Abbreviations: CRC, colorectal cancer; FIT, fecal immunochemical test; mt-sDNA, multitarget stool DNA

**Supplemental figure 10. Generalizability of findings for individuals at average risk.** The overall sample was re-weighted to match the composition of the 2019 US Census estimates in terms of gender, age, race, and ethnicity. Bars represent estimated mean marginal utilities and corresponding 95% confidence interval. Abbreviation: SD, standard deviation

# Supplemental tables

**Supplemental table 1. Sample quotas**

| **Characteristic** | **Quota** | |
| --- | --- | --- |
|  | **Lower bound** | **Upper bound** |
| **Race** |  |  |
| White | 78% | 82% |
| Non-white | 10% | 14% |
| Asian or Asian-American | 4% | 8% |
| **Ethnicity** |  |  |
| Hispanic | 11% | 15% |
| Non-Hispanic | 89% | 85% |
| **Gender** |  |  |
| Male | 40% | 50% |
| Female | 40% | 50% |
| Other | 0% | 2% |
| **Age** |  |  |
| 45–49 years | 30% | 40% |
| 50–54 years | 12% | 16% |
| 55–59 years | 12% | 16% |
| 60–64 years | 12% | 16% |
| 65–69 years | 12% | 16% |
| 70–75 years | 12% | 16% |

Demographic recruitment quotas were defined based on the 2019 US Census, but individuals aged 45 to 49 years were strategically oversampled to facilitate subgroup analysis for this age group due to divergence in current guidelines.

**Supplemental table 2. DCE internal validity testing**

|  | **Individuals at average risk  (n=1,249)** | | | **Physicians  (n=400)** | |
| --- | --- | --- | --- | --- | --- |
| **Dominance Test** | **N** | **%** | **N** | | **%** |
| Fail | 79 | (6.3) | 9 | | (2.3) |
| Pass | 1170 | (93.7) | 391 | | 97.8) |
| **Repeated Questions** | | | | | |
| Inconsistent | 224 | (17.9) | 40 | | (10.0) |
| Same answers in both | 1025 | (82.1) | 360 | | (90.0) |
| **Always Choosing A or B** | | | | | |
| Always trading | 1238 | (99.1) | 400 | | (100.0) |
| Always choosing A | 10 | (0.8) | 0 | | (0.0) |
| Always choosing B | 1 | (0.1) | 0 | | (0.0) |
| **Lexicographic preferences** |  |  |  | |  |
| Not choosing based on single attribute | 1111 | (89.0) | 246 | | (61.5) |
| Always choosing alternative better on type of test | 0 | (0.0) | 0 | | (0.0) |
| Always choosing alternative better on frequency of test | 38 | (3.0) | 1 | | (0.3) |
| Always choosing alternative better on true-positive rate | 100 | (8.0) | 44 | | (11.0) |
| Always choosing alternative better on true-negative rate | 0 | (0.0) | 0 | | (0.0) |
| Always choosing alternative better on adenoma true-positive rate | NA | NA | 109 | | (27.3) |
| **Time to Complete Survey (in minutes)** | | | | | |
| 5.00 to 6.59 | 42 | (3.4) | 34 | | (8.5) |
| 7.00 to 9.59 | 177 | (14.2) | 68 | | (17.0) |
| 10.00 to 14.59 | 366 | (29.3) | 96 | | (24.0) |
| 15.00 to 19.59 | 243 | (19.5) | 59 | | (14.8) |
| 20.00 to 29.59 | 224 | (17.9) | 49 | | (12.3) |
| 30.00 to 39.59 | 73 | (5.8) | 16 | | (4.0) |
| 40.00 to 49.59 | 29 | (2.3) | 13 | | (3.3) |
| 50.00 to 59.59 | 22 | (1.8) | 6 | | (1.5) |
| ≥60 | 73 | (5.8) | 59 | | (14.8) |

**Supplemental table 3. Mixed logit model of individuals at average risk (IAR) cohort**

|  | IAR cohort (n=1,249) | | | | | |
| --- | --- | --- | --- | --- | --- | --- |
| **Attributes** | **Mean** | **SE** | **95% CI** | **SD** | **SE** | **95% CI** |
| **Alternative-specific constant** |  |  |  |  |  |  |
| Option A |  |  |  |  |  |  |
| Option B | -0.094^***^ | (0.026) | [-0.145; -0.043] |  |  |  |
| **Type of test** |  |  |  |  |  |  |
| Colonoscopy | REF | REF | REF | REF | REF | REF |
| Blood test | 0.190^***^ | (0.051) | [0.091; 0.290] | 0.605^***^ | (0.080) | [0.448; 0.761] |
| At-home stool-based test | 0.139^**^ | (0.055) | [0.031; 0.247] | 0.885^***^ | (0.068) | [0.752; 1.018] |
| **Frequency of test** |  |  |  |  |  |  |
| Every 10 years | 0.000 | (0.000) | [0.000; 0.000] | 0.000 | (0.000) | [0.000; 0.000] |
| Every 3 years | 0.175^***^ | (0.042) | [0.093; 0.258] | 0.354^***^ | (0.111) | [0.136; 0.572] |
| Every year | 0.264^***^ | (0.052) | [0.161; 0.366] | 0.844^***^ | (0.074) | [0.699; 0.990] |
| **True positive rate** |  |  |  |  |  |  |
| 6 out of 10 (60%) *(ref)* | 0.000 | (0.000) | [0.000; 0.000] | 0.000 | (0.000) | [0.000; 0.000] |
| 7 out of 10 (70%) | 0.788^***^ | (0.057) | [0.677; 0.900] | 0.182 | (0.215) | [-0.240; 0.603] |
| 8 out of 10 (80%) | 1.517^***^ | (0.066) | [1.388; 1.646] | 0.005 | (0.117) | [-0.224; 0.234] |
| 9 out of 10 (90%) | 2.080^***^ | (0.074) | [1.934; 2.226] | 0.342^***^ | (0.124) | [0.100; 0.584] |
| 10 out of 10 (100%) | 2.698^***^ | (0.092) | [2.517; 2.878] | 1.008^***^ | (0.084) | [0.844; 1.172] |
| **True negative rate** |  |  |  |  |  |  |
| 7 out of 10 (70%) *(ref)* | 0.000 | (0.000) | [0.000; 0.000] | 0.000 | (0.000) | [0.000; 0.000] |
| 8 out of 10 (80%) | 0.651^***^ | (0.044) | [0.564; 0.738] | 0.011 | (0.086) | [-0.158; 0.179] |
| 9 out of 10 (90%) | 1.122^***^ | (0.054) | [1.016; 1.228] | 0.489^***^ | (0.078) | [0.337; 0.642] |
| 10 out of 10 (100%) | 1.540^***^ | (0.068) | [1.406; 1.675] | 1.000^***^ | (0.083) | [0.838; 1.162] |
| **Log-likelihood** | -6984 |  |  |  |  |  |
| **AIC** | 14013 |  |  |  |  |  |
| **BIC** | 14184 |  |  |  |  |  |
| **McFadden Adjusted R^2^** | 0.196 |  |  |  |  |  |

*** p<0.001, ** p<0.01, * p<0.05
Abbreviations: AIC, Akaike information criterion; BIC, Bayesian information criterion; CI, confidence interval; REF, reference; SD, standard deviation; SE, standard error

**Supplemental table 4. Weighted mixed logit model of individuals at average risk (IAR) cohort**

|  | IAR cohort (n=1,249) | | | | | |
| --- | --- | --- | --- | --- | --- | --- |
| **Attributes** | **Mean** | **SE** | **95% CI** | **SD** | **SE** | **95% CI** |
| **Alternative specific constant** |  |  |  |  |  |  |
| Option A |  |  |  |  |  |  |
| Option B | -0.117^***^ | (0.026) | [-0.168; -0.066] |  |  |  |
| **Type of test** |  |  |  |  |  |  |
| Colonoscopy | REF | REF | REF | REF | REF | REF |
| Blood test | 0.180^***^ | (0.050) | [0.081; 0.279] | 0.579^***^ | (0.082) | [0.419; 0.739] |
| At-home stool-based test | 0.143^***^ | (0.055) | [0.034; 0.251] | 0.913^***^ | (0.067) | [0.780; 1.045] |
| **Frequency of test** |  |  |  |  |  |  |
| Every 10 years | REF | REF | REF | REF | REF | REF |
| Every 3 years | 0.215^***^ | (0.042) | [0.133; 0.297] | 0.286^**^ | (0.132) | [0.027; 0.545] |
| Every year | 0.255^***^ | (0.053) | [0.152; 0.358] | 0.884^***^ | (0.074) | [0.739; 1.029] |
| **True-positive rate** |  |  |  |  |  |  |
| 6 out of 10 (60%) | REF | REF | REF | REF | REF | REF |
| 7 out of 10 (70%) | 0.813^***^ | (0.057) | [0.701; 0.925] | 0.192 | (0.211) | [-0.222; 0.607] |
| 8 out of 10 (80%) | 1.493^***^ | (0.065) | [1.366; 1.621] | 0.003 | (0.106) | [-0.205; 0.211] |
| 9 out of 10 (90%) | 2.078^***^ | (0.074) | [1.932; 2.224] | 0.316^**^ | (0.134) | [0.053; 0.579] |
| 10 out of 10 (100%) | 2.754^***^ | (0.093) | [2.570; 2.937] | 1.036^***^ | (0.084) | [0.872; 1.201] |
| **True negative** |  |  |  |  |  |  |
| 7 out of 10 (70%) | REF | REF | REF | REF | REF | REF |
| 8 out of 10 (80%) | 0.592^***^ | (0.044) | [0.505; 0.679] | 0.016 | (0.091) | [-0.163; 0.196] |
| 9 out of 10 (90%) | 1.076^***^ | (0.054) | [0.971; 1.182] | 0.527^***^ | (0.076) | [0.379; 0.676] |
| 10 out of 10 (100%) | 1.514^***^ | (0.067) | [1.382; 1.646] | 0.920^***^ | (0.083) | [0.758; 1.082] |
| **Log-likelihood** | -6955 |  |  |  |  |  |
| **AIC** | 13957 |  |  |  |  |  |
| **BIC** | 14128 |  |  |  |  |  |
| **McFadden Adjusted R^2^** | 0.199 |  |  |  |  |  |

The overall sample was re-weighted to match the composition of the 2019 US Census estimates in terms of gender, age, race, and ethnicity. *** p<0.001, ** p<0.01, * p<0.05
Abbreviations: AIC, Akaike information criterion; BIC, Bayesian information criterion; CI, confidence interval; REF, reference; SD, standard deviation; SE, standard error

**Supplemental table 5. Mixed logit model of physician cohort**

|  | Physician (n=400) | | | | | |
| --- | --- | --- | --- | --- | --- | --- |
| **Attributes** | **Mean** | **SE** | **95% CI** | **SD** | **SE** | **95% CI** |
| **Alternative specific constant** |  |  |  |  |  |  |
| Option A |  |  |  |  |  |  |
| Option B | -0.010 | (0.063) | [-0.135 - 0.114] |  |  |  |
| **Type of test** |  |  |  |  |  |  |
| Colonoscopy | 0.000 | (0.000) | [0.000; 0.000] | 0.000 | (0.000) | [0.000 ;0.000] |
| Blood test | -0.475^***^ | (0.126) | [-0.723; -0.227] | 0.541^***^ | (0.186) | [0.177; 0.905] |
| At-home stool-based test | -0.582^***^ | (0.136) | [-0.848; -0.315] | 0.428^*^ | (0.234) | [-0.030; 0.887] |
| **Frequency of test** |  |  |  |  |  |  |
| Every 10 years | 0.000 | (0.000) | [0.000; 0.000] | 0.000 | (0.000) | [0.000; 0.000] |
| Every 3 years | 0.151 | (0.116) | [-0.076; 0.378] | 0.038 | (0.182) | [-0.319; 0.395] |
| Every year | 0.162 | (0.129) | [-0.092; 0.415] | 0.020 | (0.427) | [-0.817; 0.858] |
| **True-positive rate** |  |  |  |  |  |  |
| 6 out of 10 (60%) | 0.000 | (0.000) | [0.000; 0.000] | 0.000 | (0.000) | [0.000; 0.000] |
| 7 out of 10 (70%) | 1.387^***^ | (0.148) | [1.097; 1.677] | 0.545^**^ | (0.225) | [0.104; 0.986] |
| 8 out of 10 (80%) | 2.195^***^ | (0.168) | [1.866; 2.524] | 0.329 | (0.265) | [-0.190; 0.848] |
| 9 out of 10 (90%) | 3.276^***^ | (0.197) | [2.891; 3.661] | 0.295 | (0.357) | [-0.406; 0.995] |
| 10 out of 10 (100%) | 4.208^***^ | (0.242) | [3.733; 4.682] | 0.954^***^ | (0.197) | [0.567; 1.341] |
| **True negative** |  |  |  |  |  |  |
| 7 out of 10 (70%) | 0.000 | (0.000) | [0.000; 0.000] | 0.000 | (0.000) | [0.000; 0.000] |
| 8 out of 10 (80%) | 0.027 | (0.111) | [-0.191; 0.244] | 0.090 | (0.331) | [-0.559; 0.739] |
| 9 out of 10 (90%) | 0.782^***^ | (0.118) | [0.552; 1.013] | 0.258 | (0.385) | [-0.496; 1.011] |
| 10 out of 10 (100%) | 0.912^***^ | (0.125) | [0.667; 1.157] | 0.583^***^ | (0.181) | [0.228; 0.938] |
| **Adenoma-true-positive rate** |  |  |  |  |  |  |
| 2 out of 10 (20%) *(ref)* | 0.000 | (0.000) | [0.000; 0.000] | 0.000 | (0.000) | [0.000; 0.000] |
| 5 out of 10 (50%) | 1.238^***^ | (0.114) | [1.014; 1.462] | 0.019 | (0.160) | [-0.296; 0.333] |
| 10 out of 10 (100%) | 4.127^***^ | (0.234) | [3.668; 4.586] | 1.916^***^ | (0.152) | [1.617; 2.214] |
| **Log-likelihood** | -1456 |  |  |  |  |  |
| **AIC** | 2966 |  |  |  |  |  |
| **BIC** | 3141 |  |  |  |  |  |
| **McFadden Adjusted R^2^** | 0.571 |  |  |  |  |  |

*** p<0.001, ** p<0.01, * p<0.05
Abbreviations: AIC, Akaike information criterion; BIC, Bayesian information criterion; CI, confidence interval; SE, standard error

**Supplemental table 6. Average partial effect for IAR and physician cohorts**

| Attributes | General Population | |  | Physicians | |
| --- | --- | --- | --- | --- | --- |
|  | **APE (SE)** | **[95% CI]** |  | **APE (SE)** | **[95% CI]** |
| **Type** |  |  |  |  |  |
| Colonoscopy | REF | REF |  | REF | REF |
| Blood test | 0.034 (0.009) | [0.016; 0.052] |  | -0.050 (0.013) | [-0.077; -0.024] |
| At-home stool-based test | 0.025 (0.010) | [0.006; 0.044] |  | -0.062 (0.014) | [-0.090; -0.034] |
| **Frequency** |  |  |  |  |  |
| Every 10 years | REF | REF |  | REF | REF |
| Every 3 years | 0.031 (0.008) | [0.017; 0.046] |  | 0.016 (0.012) | [-0.008; 0.040] |
| Every year | 0.047 (0.009) | [0.029; 0.065] |  | 0.017 (0.014) | [-0.010; 0.044] |
| **True positive** |  |  |  |  |  |
| 60% | REF | REF |  | REF | REF |
| 70% | 0.134 (0.009) | [0.116; 0.151] |  | 0.142 (0.014) | [0.114; 0.200] |
| 80% | 0.273 (0.009) | [0.255; 0.292] |  | 0.233 (0.014) | [0.206; 0.300] |
| 90% | 0.381 (0.009) | [0.363; 0.399] |  | 0.355 (0.013) | [0.329; 0.400] |
| 100% | 0.487 (0.010) | [0.467; 0.508] |  | 0.453 (0.015) | [0.424; 0.500] |
| **True negative** |  |  |  |  |  |
| 70% | REF | REF |  | REF | REF |
| 80% | 0.117 (0.007) | [0.103; 0.132] |  | 0.003 (0.012) | [-0.020; 0.026] |
| 90% | 0.203 (0.008) | [0.187; 0.219] |  | 0.083 (0.012) | [0.060; 0.106] |
| 100% | 0.277 (0.010) | [0.258; 0.296] |  | 0.097 (0.013) | [0.072; 0.122] |
| **Adenoma true-positive rate** |  |  |  |  |  |
| 20% | – | – |  | REF | REF |
| 50% | – | – |  | 0.142 (0.011) | [0.120; 0.164] |
| 100% | – | – |  | 0.462 (0.011) | [0.441; 0.483] |

Abbreviations: APE, average partial effect; CI, confidence interval; REF, reference; SE, standard error

**Supplemental table 7. Screening test preferences of individuals at average risk based on age, race, and prior screening experience**

|  | **FIT** | |  | **mt-sDNA** | |  | **Blood test** | |  | **Colonoscopy** | |
| --- | --- | --- | --- | --- | --- | --- | --- | --- | --- | --- | --- |
| **Characteristic** | **Uptake  (SE)** | **[95% CI]** |  | **Uptake  (SE)** | **[95% CI]** |  | **Uptake  (SE)** | **[95% CI]** |  | **Uptake  (SE)** | **[95% CI]** |
| **Age** |  |  |  |  |  |  |  |  |  |  |  |
| 45 to 49 (n=305) | 20.9^***^ (1.5) | [17.9; 23.9] |  | 36.5^***^ (1.8) | [33.0; 40.0] |  | 12.0^***^ (1.1) | [9.8; 14.1] |  | 30.6^***^ (1.6) | [27.5; 33.8] |
| 50 to 64 (n=538) | 19.8^***^ (1.2) | [17.4; 22.2] |  | 38.7^***^ (1.6) | [35.5; 41.9] |  | 9.5^***^ (0.8) | [8.0; 11.1] |  | 32.0^***^ (1.5) | [29.1; 34.9] |
| 65 to 75 (n=406) | 17.3^***^ (1.3) | [14.8; 19.7] |  | 40.4^***^ (1.7) | [37.0; 43.8] |  | 7.8^***^ (0.7) | [6.3; 9.2] |  | 34.5^***^ (1.6) | [31.4; 37.7] |
| Race |  |  |  |  |  |  |  |  |  |  |  |
| White (n=1,021) | 18.0^***^ (0.9) | [16.2; 19.9] |  | 39.7^***^ (1.6) | [36.6; 42.7] |  | 8.8^***^ (0.6) | [7.6; 10.0] |  | 33.5^***^ (1.4) | [30.7; 36.3] |
| Non-white (n=228) | 24.9^***^ (1.6) | [21.7; 28.1] |  | 34.8^***^ (1.9) | [31.2; 38.5] |  | 12.6^***^ (1.1) | [10.5; 14.7] |  | 27.6^***^ (1.6) | [24.5; 30.8] |
| Prior screening |  |  |  |  |  |  |  |  |  |  |  |
| Never (n=526) | 20.7^***^ (1.1) | [18.6; 22.7] |  | 41.9^***^ (1.8) | [38.4; 45.4] |  | 9.6^***^ (0.8) | [8.1; 11.1] |  | 27.8^***^ (1.7) | [24.5; 31.1] |
| Non-invasive^†^ (n=106) | 26.1^***^ (1.4) | [23.3; 28.9] |  | 52.8^***^ (2.4) | [48.1; 57.5] |  | 7.2^***^ (1.1) | [5.0; 9.3] |  | 14.0^***^ (2.1) | [9.7; 18.2] |
| Invasive^†^ (n=617) | 16.4^***^ (0.9) | [14.6; 18.3] |  | 33.3^***^ (1.6) | [30.1; 36.4] |  | 9.2^***^ (0.7) | [7.8; 10.6] |  | 41.1^***^ (1.9) | [37.3; 44.9] |

^†^ Blood and stool tests considered non-invasive; colonoscopy and flexible sigmoidoscopy considered as invasive
Abbreviations: CI, confidence interval; FIT, fecal immunochemical test; mt-sDNA, multitarget stool DNA; SE, standard error
